# The use of Oxford Nanopore native barcoding for complete genome assembly

**DOI:** 10.1093/gigascience/gix001

**Published:** 2017-02-24

**Authors:** Sion C Bayliss, Vicky L Hunt, Maho Yokoyama, Harry A Thorpe, Edward J Feil

**Affiliations:** The Milner Centre for Evolution, Department of Biology and Biochemistry, University of Bath, Claverton Down, Bath BA2 7AY, UK

**Keywords:** Whole genome sequencing, *Staphylococcus aureus*, MinION, long read, hybrid assembly, bacterial genomics, multiplexing, native barcoding

## Abstract

**Background:**

The Oxford Nanopore Technologies MinION(TM) is a mobile DNA sequencer that can produce long read sequences with a short turn-around time. Here we report the first demonstration of single contig genome assembly using Oxford Nanopore native barcoding when applied to a multiplexed library of 12 samples and combined with existing Illumina short read data. This paves the way for the closure of multiple bacterial genomes from a single MinION(TM) sequencing run, given the availability of existing short read data. The strain we used, MHO_001, represents the important community-acquired methicillin-resistant *Staphylococcus aureus* lineage USA300.

**Findings:**

Using a hybrid assembly of existing short read and barcoded long read sequences from multiplexed data, we completed a genome of the *S. aureus* USA300 strain MHO_001. The long read data represented only ∼5% to 10% of an average MinION(TM) run (∼7x genomic coverage), but, using standard tools, this was sufficient to complete the circular chromosome of *S. aureus* strain MHO_001 (2.86 Mb) and two complete plasmids (27 Kb and 3 Kb). Minor differences were noted when compared to USA300 reference genome, USA300_FPR3757, including the translocation, loss, and gain of mobile genetic elements.

**Conclusion:**

Here we demonstrate that MinION(TM) reads, multiplexed using native barcoding, can be used in combination with short read data to fully complete a bacterial genome. The ability to complete multiple genomes, for which short read data is already available, from a single MinION(TM) run is set to impact our understanding of accessory genome content, plasmid diversity, and genome rearrangements.

## Introduction

The spread of methicillin-resistant *Staphylococcus aureus* represents a significant burden in both the health-care setting and the community. The USA300 clone is a particular cause for concern, being responsible for an increasing number of skin and soft-tissue infections within the community, particularly in North America [1]. The advent of new sequencing technologies is set to inform on novel intervention and surveillance strategies, although important technical limitations remain. Whilst short read data provide an excellent means to assay the variation within the core genome, which is useful for reconstructing hospital outbreaks, it is usually not possible to infer genome rearrangements or to fully assemble mobile genetic elements such as plasmids from these data. Closure of bacterial genomes has been demonstrated on *Escherichia coli* using the Oxford Nanopore Technologies (ONT) MinION(TM) reads alone and on a range of bacteria including *Bacteriodes fragilis, Acinetobacter baylyi*, and *Francisella* spp. using a hybrid approach combining error-prone long reads with low error rate short reads [2–5]. Here we demonstrate that it is also possible to generate complete genomes using multiplexed reads from a single MinION(TM) run in combination with matched Illumina short reads. We used a strain of *S. aureus* of the USA300 lineage as an example.

## Methods

### MinION(TM) library construction and sequencing

*S. aureus* strain MHO_001 was recovered in 2015 from asymptomatic nasal carriage via a standard nasal swab of a healthy individual with informed consent. DNA from an overnight culture was extracted using the Qiagen Genomic Tip 500/G Kit, following the manufacturer's instructions, except lysozyme was replaced with lysostaphin to a final concentration of 200 μg/ml. Sequencing library preparation was carried out with Nanopore Genomic Sequencing Kit SQK-MAP006 (ONT, UK) and a PCR-free ‘native barcoding’ kit provided by ONT. The NEBNext Ultra II End Repair/dA Tailing kit (E7546S, NEB) was used to prepare 1000 ng of sheared genomic DNA (1000 ng DNA in 50 μl nuclease free water, 7 μl of Ultra II End-Prep Buffer, 3 μl Ultra II End-Prep Enzyme Mix in a total volume of 60 μl). The reaction was incubated for 5 minutes at 20°C and heat inactivated for 5 minutes at 65°C. The DNA was purified using a 1:1 volume of Agencourt AMPure XP beads (A63880, Beckman Coulter) according to manufacturer's instructions and eluted in 31 μl of nuclease free water. Blunt/TA Ligase Master Mix (M0367S, NEB) was used to ligate native barcode adapters to 22.5 μl of 500 ng end prepared DNA for 10 minutes at room temperature. The barcoded DNA was purified using a 1:1 volume of AMPure XP beads and eluted in 26 μl nuclease free water. Twelve barcoded samples from diverse sources including other bacterial samples were pooled, 58 ng of each sample was added to give 700 ng of pooled library DNA. Hairpin adapters were ligated using 10 μl Native Barcoding Adapter Mix, 50 μl Blunt/TA Ligase Master Mix, and 2 μl Native Barcoding Hairpin Adapter added to 38 μl of the pooled library DNA to give a final reaction volume of 100 μl. The reaction mixture was incubated for 10 minutes at room temperature before the addition of 1 μl of HP tether and a further 10 minutes incubation. The final reaction was cleaned using prewashed Dynabeads MyOne Streptavidin C1 beads (65001; Thermo Fisher Scientific). DNA concentrations at each step were measured using a Qubit Fluorometer. Then 6 μl of the pooled, barcoded library was mixed with 65 μl nuclease free water, 75 μl 2x Running Buffer, and 4 μl Fuel Mix (SQK-MAP006, ONT) and immediately loaded onto a MinION(TM) Flow Cell Mk I R7.3 on a MinION(TM) MkI controlled by MinKNOW version 0.50.2.15 software (ONT). Base calling was performed using Metrichor ONT Sequencing Workflow Software v1.19.0 with the Basecall_Barcoding workflow (ONT). The additional DNA samples included in the pooled library were a diverse assemblage of bacterial and eukaryotic DNA samples provided by attendees during the PoreCamp Workshop 2015 at the University of Birmingham. The additional pooled library samples are being prepared for separate publication. Details on the PoreCamp Workshop and associated publications can be found at http://porecamp.github.io/. MinION reads were deposited in the European Nucleotide Archive under study accession PRJEB14152.

### Illumina library construction and sequencing

An overnight culture was grown on TSB agar from a 15% glycerol stock maintained at −80°C. An aliquot of the culture was added to tubes containing DNA beads and library preparation was carried out by MicrobesNG, University of Birmingham (http://microbesng.uk). A single 250-bp paired end library was constructed and sequenced on both MiSeq and HiSeq Illumina platforms. The reads from both sequencing runs were combined before downstream analysis. The sequenced strain is stored in the MicrobesNG indexed repository as strain 2998-174. Reads were deposited in the European Nucleotide Archive under study accession PRJEB14152.

### Assembly, annotation, and analysis

**Figure 1.**
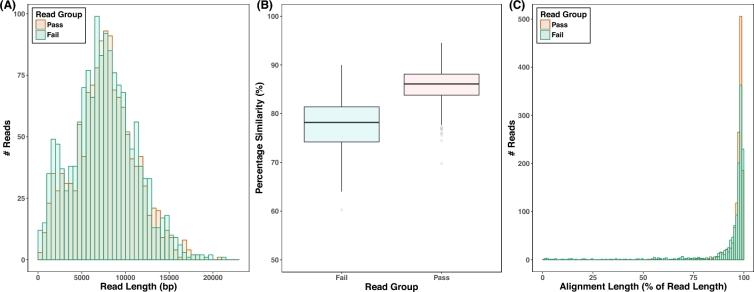
Figure summarizing read statistics for the 2D nanopore pass (red) and fail (green) reads. (A) Read length distributions of pass and fail reads. Data were binned every 500 bp. (B) Box and whisker plot of the sequence similarity of nanopore reads to the genome of MHO_001 as determined by BLASR. Only the alignment with the highest percentage similarity was considered for each read. The lower and upper “hinges” correspond to the first and third quartiles. The upper and lower whiskers extend from the hinge to the most extreme value that is within 1.5× interquartile range. Data beyond the end of the whiskers are outliers and plotted as points. (C) The distribution of BLASR alignment lengths of nanopore reads as a percentage of the original read length. Only the alignment with the highest percentage similarity was considered for each read. Nanopore 2D reads with a phred score >8 were classified by Metrichor as pass reads (blue), and all other 2D reads were classified as fail reads (blue).

The full informatics analysis and associated data are available as a step-by-step walk-through at https://github.com/SionBayliss/MHO_analysis. Illumina reads were trimmed using Trimmomatic-0.33 [6]. Reads were trimmed to a minimum read quality of Q15. Reads <30 bp in length were excluded and sequencing adapters were removed. MinION(TM) 2D reads were filtered into pass and fail reads by the Metrichore basecaller; hereafter, these two categories of reads will be referred to as “2D pass” and “2D fail” reads, following the terminology adopted by the manufacturer and used in Karlsson et al. and Ip et al. [4, 7]. These are equivalent to the “high quality” and “low quality” read groups from Oikonomopoulos et al. [8]. MinION(TM) 1D reads were not used for this analysis. The 2D fail reads, those which did not pass the basecaller quality threshold, were demultiplexed using an in-house script (FilterBarcodes.pl). The twelve 40-bp barcodes used for library construction were compared in a moving 40-bp window to the sequence in the first and last 150 bp of each read. The barcode requiring the fewest insertions, deletions, or substitutions to be permuted into a sequence in the beginning or end of a read, with a maximum cut-off of 14 permutations, was considered a match. Each read could be assigned to only one individual sample; in the case of a tie the reads were discarded. Sequence preceding or following the presence of a barcode at the beginning or end or a read, respectively, were trimmed as adapter sequence. After quality trimming, 439,480 paired short reads, 1324 2D pass reads, and 1499 demultiplexed 2D fail reads (2823 total) nanopore long reads were passed as input files to SPAdes v3.6.1 using the –nanopore, –cov-cutoff 5, and –careful –options [9]. The nanopore reads had a median read length of 7577 bp, a maximum length of 23,380 bp, and a minimum length of 250 bp (Fig. 1A). After assembly, all contigs <300 bp were removed. This resulted in three contigs, the complete chromosome of MHO_001, and two complete plasmids. The contigs were circularized by MUSCLE v3.8.31 alignment (default parameters) of identical overlapping regions at the end of contigs and removal of one alternative overlapping sequence using an in-house script (CirculariseOnOverlaps.pl) [10]. Start sites were fixed relative to the beginning of the relevant reference sequence. A BLAST search against the nt/nr database using default megablast settings revealed the closest, well studied, reference genome was USA300_FPR3757 (Genbank:CP000255) [11]. The two smaller contigs were 100% identical in both aligned sequence and alignment length to previously sequenced *S. aureus* lineage USA300 plasmids, SAP046A (Genbank:GQ900404.1) and SAP046B (Genbank:GQ900403.1). The smallest plasmid was also identical to USA300_FPR3757 plasmid pUSA01 (CP000256). The complete genome of MHO_001 was annotated using Prokka 1.11 [12].

To calculate per base read coverage, short and long reads were mapped to MHO_001 using BWA 0.7.12-r1039 and coverage was calculated using samtools 1.2 [13, 14]. Nanopore reads were mapped using the ‘bwa mem -x ont2d’ option. To assess the sequence similarity and number of reads mapped between the long reads and the MHO_001 assembly, the nanopore 2D pass, demultiplexed 2D fail reads, and 2D fail reads in which no barcodes were identified were aligned to the MHO_001, including plasmids, using BLASR (Fig. 1, Table 1) [15]. SNPs were called between the chromosome and reference genome using MAUVE [16]. SNPs were further confirmed by mapping short reads independently to USA300_FPR3757 and calling variants. Mapping was performed using BWA, reads at indel sites were realigned using the GATK toolbox and SNPs were called using samtools [14, 17]. The variant call file was filtered for variants supported by a minimum read depth of 4 (minimum 2 per strand), >30 map quality, >50 average base quality, no significant strand bias, and >75% of reads supporting the variant. Indels were additionally confirmed using pindel [18]. The variant call file was filtered to remove regions unique to MHO_001 or USA300_FPR3757. Repeat regions of >50 bp, which are notoriously problematic for short read mapping, were identified using nucmer and removed from the comparison [19] (Supplementary Table 1). The absence of SAPI5 in MHO_001 and expansion of the tRNA island at 554,826 were confirmed using PCR and Sanger sequencing (Supplementary Analysis).

**Table 1.** Table summarizing the BLASR analysis of semultiplexed 2D pass and fail nanopore long reads assigned to sample MHO_001. Reads were aligned to the assembled MHO_001 reference genome using BLASR with default parameters. Only the alignment with the highest percentage similarity was considered for each read. The average alignment length was calculated from the length of the top BLASR alignment relative to the length of the input read

	Pass	Fail
# Reads	1324	1499
# BLASR hits (% # reads)	1320 (99.70)	1292 (86.19)
Mean alignment length (%)	96.79	92.90
Mean similarity (%)	85.87	77.76
# Hits <75% read length (%)	11 (0.83)	93 (7.20)
# Hits ≧75% read length (%)	1309 (99.17)	1199 (92.80)

## Results and discussion

A hybrid assembly using a low coverage of MinION(TM) reads (6–8×) combined with moderate coverage Illumina reads (∼50×) was used to generate a complete genome. The assembly resolved regions of the genome that were problematic for short read assembly alone, such as chromosomal rRNA operons. The generation of a complete genome from only ∼5% of the possible current yield of a MinION(TM) run using a multiplexed library should represent a cost-effective means to complete multiple genomes during a single MinION(TM) sequencing run, although the approach also requires matching short read Illumina data. Larger or more complex bacterial genomes may require higher coverage read data alongside additional bioinformatics analyses to generate comparably polished, complete genomes [3].

**Figure 2. fig2:**
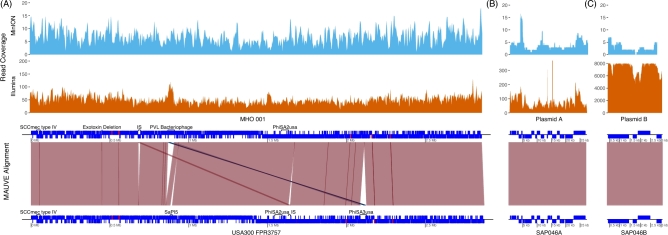
Alignment of MHO_001 chromosome (A), plasmid A (B), and plasmid B (C) to the USA300_FPR3757 genome and reference plasmids alongside long and short read coverage. The bottom panels show alignments between MHO_001 and the reference sequences. Contiguous sequences are shown by connecting red lines and inversions are depicted in blue. Coding sequences (CDS) are annotated as blue rectangles with the exception of ribosomal RNA operons, which are represented by red rectangles. Those above the line represent open reading frames on the forward strand and those under the line on the reverse strand. Notable mobile genetic elements or genomic features are annotated. A scale bar in bp is presented underneath each contig. The middle panels represent per base read coverage of short reads across the MHO_001 genome. The data was binned every 1000 bp. The y-axis, representing per bin read coverage, has been constrained to 200, 350, and 8000 reads per bin for the MHO_001 chromosome, plasmid A, and plasmid B, respectively. The top panel represents the per base read coverage of nanopore long reads across the MHO_001 genome. The data was binned every 1000 bp. The y-axis, representing per bin read coverage, has been constrained to 20 reads per bin for each contig.

By demultiplexing the 2D fail reads, we were able to double the number of nanopore reads for assembly from 1324 to 2823 reads. The nanopore reads were aligned to the complete MHO_001 genome using BLASR (Fig. 1, Table 1). 1320/1324 (99.7%) 2D pass reads demultiplexed by Metrichor aligned to the assembly with an average percentage similarity of 85.9% and a mean alignment length of 96.8% of the input read. 1292/1499 (99.7%) 2D fail reads demultiplexed by in-house scripts aligned to the assembly with an average percentage similarity of 77.76% and a mean alignment length of 92.9%. The fail reads in which we failed to find a barcode contained 722/9501 (7.6%) reads[Fig fig2] that aligned to the MHO_001 genome. In summary, a considerable amount of useful information was contained within the demultiplexed 2D fail reads without which we would have been unable to produce a complete genome. We can conclude that we were able to correctly identify the ONT barcodes in ∼85% of the 2D fail reads used for assembly.

The chromosome showed minor differences to the USA300 reference genome USA300_FPR3757 including 155 SNP differences and the loss and gain of mobile genetic elements (Fig. 2). To provide an independent confirmation of the 155 SNP differences identified by MAUVE between aligned regions of MHO_001 and USA300_FPR3757, the short reads were mapped to USA300_FPR3757 and variants were called using strict parameters. Of the 155 MAUVE SNPs, 41 (26.5%) were present in repeat regions and excluded from the comparison. Of the remaining 114 SNPs, 111 (97.4%) were supported by short read mapping to USA300_FPR3757. The remaining 3 SNPs (2.6%) were unsupported. No indels were identified by short read mapping to MHO_001 by either GATK/samtools or pindel. In summary, of the 114 SNPs identified by MAUVE that could be robustly investigated by short read mapping, 111 (97.4%) were confirmed using low error rate short reads. Furthermore, the long and short read coverage support at the edge of each of the large structural variants in MHO_001 was 8 to 10x for nanopore reads, with the exception of the 3^΄^ edge of the transposed 13,356-bp insertion sequence, which had a read coverage of 3x compared to the genomic average of 6.8x coverage. The edge of each structural variant was supported by >25 short reads.

There was minor sequence dissimilarity, including a small deletion, in ribosomal RNA operons. This could either reflect evolutionary changes in these highly conserved sequences or minor misassembly; these regions are typically difficult to assemble. MHO_001 lacked Staphylococcal pathogenicity island 5 (SAPI5), a 13,960-bp exotoxin encoding transposon observed at position 881,852 in the reference. MHO_ also lacked the prophage phiSA3USA, which harbours the important virulence factor staphylokinase. As the integration site of this phage (the *hlb* gene) is intact, it is possible that MHO_001 has never acquired this phage. MHO_001 contained a 42,297-bp tyrosine recombinase bacteriophage integrated at position 867,385. This bacteriophage contained a beta-lactamase and a putative Panton-Valentine-like leuckocidin and several hypothetical genes. The position of an insertion sequence containing ftsK translocase differs between MHO_001 and the reference genome, consistent with a translocation event (USA300_FPR3757:1,630,720-1,644,076 to MHO_001:679,522-692,877). The location of this element in MHO_001 truncates a gene of unknown function. There is a short 1282-bp deletion of a gene encoding an exotoxin at position 448,767 in MHO_001. MHO_001 also has an extended tRNA cluster at 554,826 containing 7 additional tRNAs (val, thr, lys, gly, leu, arg, pro) relative to USA300_FPR3757, representing either gene expansion or reduction of this gene cluster in USA300_FPR3757.

A BLAST search revealed that the two smaller contigs were identical to previously sequenced plasmids associated with USA300 [20]. The larger of the plasmids contained an N-type replication system (repA) with a pSK1 type plasmid partitioning system. It encoded a host of resistance genotypes including macrolide (mac), erythromycin (ery), cadmium (cadX and cadD), streptothricin (sta), aminoglycoside (aad), neomycin and kanamycin (aph) resistance genes. In addition to this, the plasmid contained a Tn552-like transposon containing a beta-lactam resistance (bin, blaI, blaR1, blaZ) operon and a sin recombinase. The smaller of the two plasmids encoded three hypothetical proteins and a replicase. Both plasmids have been previously observed to occur concurrently in the same host.

There was a discrepancy observed between the coverage of short and long reads of plasmidic and chromosomal contigs (Fig. 2, top and middle panels). The average chromosomal coverage was 49.6x (7.0 SD) with short read data and 6.8x (2.6 SD) with nanopore reads. The average short read coverage of plasmids A and B was 78.4 (8.9 SD) and 7302.0 (85.4 SD), respectively. This represents a coverage increase of 1.5- and 150-fold relative to the chromosome. The opposite trend was observed with long reads; plasmids A and B had and average coverage of 4.0 (2.0 SD) and 2.9 (1.7 SD), respectively, which represents a 40% and 60% decrease in coverage relative of the chromosome. In addition to this the smaller of the two plasmids was only intermittently covered by nanopore reads. The reduced number of mappable nanopore reads was likely due to the fragment size selection steps during library preparation. The inherent problems of aligning long error-prone reads to reference sequences may also have contributed. It is thus important that future studies attempting to reconstruct plasmids or studying plasmid diversity consider the impact of size selection on downstream analysis or to prepare multiple DNA libraries with differential size selection as previously discussed by Koren and Phillippy [21]. However, the clear benefit of hybrid sequencing is that it allows for the generation of larger assemblies with less uncertainties than by using a single sequencing technology preferentially over another.

### Additional files

Supplementary Table 1. Table summarizing the BLASR analysis of demultiplexed nontarget sample 2D nanopore long reads and 2D fail reads in which no barcode was detected. Reads were aligned to the assembled MHO_001 reference genome using BLASR with default parameters. Only the alignment with the highest percentage similarity was considered for each read. The average alignment length was calculated from the length of the top BLASR alignment relative to the length of the input read.

Supplementary Table 2. Spreadsheet summarizing the comparison between SNPs called by MAUVE alignment of assemblies created using long and short reads and SNPs called via mapping short reads to USA300_FPR3757.

Supplementary Figure 1. MAUVE alignment of the overlapping region included in the circularized single chromosomal contig aligned to USA300_FPR3757.

Supplementary Figure 2. MAUVE alignment of the overlapping region not included in the circularized single chromosomal contig aligned to USA300_FPR3757.

Supplementary Figure 3. CLUSTAL visualization of the MUSCLE alignment between the two overlapping regions at the edge of the single chromosomal contig.

Supplementary Figure 4. Tablet visualization of the nanopore long reads that span the overlapping regions at the edge of the circularized single chromosomal contig.

Supplementary Analysis. PCR and Sanger sequencing analysis of large structural variants SAPI5 and tRNA expansion.

### Competing interests

No competing interests.

### Funding

The authors would like to acknowledge BBSRC/NERC grant number BB/M026388/1 for providing funding for SB. SB and VH were also funded by a grant from the United Kingdom Clinical Research Collaboration Translational Infection Research initiative, and the Medical Research Council (Grant Number G1000803, held by Prof. Sharon Peacock) with contributions from the Biotechnology and Biological Sciences Research Council, the National Institute for Health Research on behalf of the Department of Health, and the Chief Scientist Office of the Scottish Government Health Directorate. The authors are grateful for travel funds provided by NERC (NE/N000501/1) for SB and Medical Research Council Cloud Infrastructure for Microbial Bioinformatics for VH to attend.

### Author contributions

SB and VH were responsible for the conception and design of study and data acquisition. SB performed the analysis and interpretation of data and manuscript drafting. MY carried out the supplementary analysis. HAT and EF revised the manuscript critically for important intellectual content. SB and EF approved the version of the manuscript to be published.

### Data availability

The dataset supporting the conclusions of this article is available in the European Nucleotide Archive repository under project number PRJEB14152. Further supporting data is also available from the *GigaScience* GigaDB repository [22].

### Availability and requirements

- Project name: MHO_001 hybrid read assembly and analysis
- Project home page: https://github.com/SionBayliss/MHO_analysis
- Operating system: Unix
- Programming language: R, perl
- Other requirements: Dependencies include Samtools (> = 1.18), Trimmomatic, SPAdes v3.6.1, BWA (0.7.5a-r405), BioPerl, MAUVE, BLASR, prokka, Tablet/Artemis
- License: GNU GPL v3

## Supplementary Material

GIGA-D-16-00028_Original_Submission.pdf

GIGA-D-16-00028_Reviewer_3.pdf

GIGA-D-16-00028_Revision_1.pdf

GIGA-D-16-00028_Revision_2.pdf

Response_to_reviewers_Orginal_Submission.pdf

Response_to_reviewer_comments_Revision_1.pdf

Response_to_reviewer_comments_Revision_2.pdf

Reviewer_1_Report_Original_Submission.pdf

Reviewer_1_Report_Revision_1.pdf

Reviewer_2_Report_Original_Submission.pdf

Reviewer_2_Report_Revision_1.pdf

Supplement Files
