# Supplementary material for: The use of Oxford Nanopore native barcoding for complete genome assembly
Source: Gigascience. 2017 Feb 24;6(3):1–6. doi: 10.1093/gigascience/gix001 (PMC5467021; doi:10.1093/gigascience/gix001)
Supplement: GIGA-D-16-00028_Original_Submission.pdf [file gix001_giga-d-16-00028_original_submission.pdf]

Manuscript
Click here to download Manuscript Bayliss_2016.pdf

The use of Oxford Nanopore native barcoding for complete genome assembly.

Sion C. Bayliss[a]*, Vicky L. Hunt[a], Harry A. Thorpe[a] and Edward J. Feil[a]

[a] The Milner Centre for Evolution, Department of Biology and Biochemistry, University of Bath, Claverton Down, Bath BA2 7AY, UK

* For correspondence: s.bayliss@bath.ac.uk. Tel: +44 (0)1225 340959

Abstract

Background

The Oxford Nanopore MinION is a mobile DNA sequencer that can produce long read sequences with a short turn-around time. Here we report the first demonstration of full genome closure using Oxford Nanopore native barcoding when applied to a multiplexed library of 12 samples and combined with existing Illumina short-read data. This paves the way for the closure of multiple bacterial genomes from a single MinION sequencing run, given the availability of existing short-read data. The strain we used, MHO_001, represents the important community-acquired methicillin resistant *Staphylococcus aureus* lineage USA300.

Findings

Using a hybrid assembly of existing short read and barcoded long read sequences from multiplexed data, we fully closed the genome of the *S. aureus* USA300 strain MHO_001. The long-read data represented only ~5-10% of an average MinION run (~7x genomic coverage), but, using standard tools, this was sufficient to close the circular chromosome of *S. aureus* strain MHO_001 (2.86 Mb) and two complete plasmids (29 Kb and 3 Kb). Minor differences were noted when compared to USA300 reference genome, USA300_FPR3757, including the translocation, loss and gain of mobile genetic elements.

Conclusion

Here we demonstrate that MinION reads, multiplexed using native barcoding, can be used in

combination with short-read data, to fully close a bacterial genome. The ability to close multiple genomes, for which short-read data is already available, from a single MinION run is set to impact on our understanding of accessory genome content, plasmid diversity and genome rearrangements.

Keywords

Whole genome sequencing, *Staphylococcus aureus*, MinION, long read, hybrid assembly, bacterial genomics, multiplexing, native barcoding.

Data description

The spread of methicillin resistant *Staphylococcus aureus* (MRSA) represents a significant healthcare burden both in health-care settings and in the community. The USA300 clone is a particular cause for concern, being responsible for an increasing number of skin and soft-tissue infections within the community, particularly in North America [1]. The advent of new sequencing technologies is set to inform on novel intervention and surveillance strategies, although important technical limitations remain. Whilst short read data provides an excellent means to assay the variation within the core genome, which is useful for reconstructing hospital outbreaks, it is usually not possible to infer genome re-arrangements or to fully assemble mobile genetic elements (MGEs) such as plasmids from these data. Closure of bacterial genomes has been demonstrated on *Escherichia coli* using MinION reads alone and on a range of bacteria including *Bacteriodes fragilis, Acinetobacter baylyi* and *Francisella* spp. using a hybrid approach combining error prone long reads with low error rate short reads [2–5]. Here we demonstrate that it is also possible to close genomes using multiplexed reads from a single MinION run in combination with matched Illumina short reads. We used a strain of *S. aureus* of the USA300 lineage as an example.

Methods

MinION library construction and sequencing

S. aureus USA300 strain MHO_001 was recovered in 2015 from a case of asymptomatic nasal carriage in the UK. DNA from an overnight culture was extracted using the Qiagen Genomic Tip 500/G Kit, following the manufacturers' instructions, except lysozyme was replaced with lysostaphin to a final concentration of 200 µg/ml. Sequencing library preparation was carried out with Nanopore Genomic Sequencing Kit SQK-MAP006 (Oxford Nanopore, UK) and a PCR free 'native barcoding' kit provided by ONT. The NEBNext Ultra II End Repair/dA Tailing kit (E7546S, NEB, USA) was used to prepare 1000 ng of sheared genomic DNA (1000 ng DNA in 50 µl nuclease free water, 7 µl of Ultra II End-Prep Buffer, 3 µl Ultra II End-Prep Enzyme Mix in a total volume of 60 ul). The reaction was incubated for 5 minutes at 20°C and heat inactivated for 5 minutes at 65°C. The DNA was purified using a 1:1 volume of Agencourt AMPure XP beads (A63880, Beckman Coulter, USA) according to manufacturer's instructions and eluted in 31 µl of nuclease free water. Blunt/TA Ligase Master Mix (M0367S, NEB, USA) was used to ligate native barcode adapters to 22.5 µl of 500ng end prepared DNA for 10 minutes at room temperature. The barcoded DNA was purified using a 1:1 volume of AMPure XP beads and eluted in 26 µl nuclease free water. Twelve barcoded samples from diverse sources including other bacterial samples were pooled, 58 ng of each sample was added to give 700 ng of pooled library DNA. Hairpin adapters were ligated using 10 µl Native Barcoding Adapter Mix, 50 µl Blunt/TA Ligase Master Mix and 2 µl Native Barcoding Hairpin Adapter (BHP) added to 38 µl the pooled library DNA to give a final reaction volume of 100 µl. The reaction mixture was incubated for 10 minutes at room temperature before the addition of 1 µl of HP tether and a further 10 min incubation. The final reaction was cleaned using pre-washed Dynabeads MyOne Streptavidin C1 beads (65001; Thermo Fisher Scientific, USA). DNA concentrations at each step were measured using a Qubit Fluorometer. 6 µl of the pooled, barcoded library was mixed with 65 µl nuclease free water, 75 µl 2x Running Buffer and 4 µl Fuel Mix (SQK-MAP006, Oxford Nanopore, UK) and immediately loaded onto an MinION. The other DNA samples included in the pooled library were a diverse assemblage of bacterial and

eukaryotic DNA samples provided by attendees during the PoreCamp Workshop 2015 at the University of Birmingham, further details of which can be found at http://porecamp.github.io/. MinION reads were deposited in the European Nucleotide Archive under accession number ERS1178418.

Illumina library construction and sequencing

An overnight culture was grown on TSB agar from a 15% glycerol stock maintained at -80 ^{o}C. An aliquot of the culture was added to tubes containing DNA beads and library preparation was carried out by MicrobesNG (http://microbesng.uk), University of Birmingham. Sequencing was performed on both MiSeq and HiSeq Illumina platforms. The sequenced strain is stored in the MicrobesNG indexed repository as strain 2998-174. Reads were deposited in the European Nucleotide Archive under accession numbers ERS1180806 and ERS1180807.

Assembly, Annotation and Analysis

The full informatics analysis and associated data is available as a step-by-step walk-through at https://github.com/SionBayliss/MHO_analysis. Illumina reads were trimmed using Trimmomatic-0.33 [6]. Reads were trimmed to a minimum read quality of Q15. Reads below 30 bp in length were excluded and sequencing adapters were removed. MinION 2D pass reads were demultiplexed by the Metrichore work flow. MinION 2D reads that failed QC were demultiplexed using splitbarcodes.py (stringency 14), followed by the removal of barcodes and adapter sequences. After trimming, 439,480 paired short reads and 3105 long reads (Median read length: 7588, Min:237, Max:23440; Figure 1) were used as an input to SPAdes using the -nanopore, --cov-cutoff 5 and --careful options [7]. Filtering all SPAdes contigs <300 bp resulted in three contigs, the complete chromosome of MHO_001, and two complete plasmids. BLAST alignment of the chromosome revealed the closest, well studied reference genome was USA300_FPR3757 (Genbank:CP000255) [8]. The two smaller contigs had 100% sequence and 100% length BLAST similarity with with previously sequenced *S. aureus* USA300 plasmids, SAP046A

(Genbank:GQ900404.1) and SAP046B (Genbank:GQ900403.1). The smallest plasmid was also identical to USA300_FPR3757 plasmid pUSA01 (CP000256). The three contigs sequences were trimmed and aligned to their closest reference sequence using progressiveMAUVE [9] and annotated using Prokka 1.11 [10].

Ilumina and Nanopore reads were mapped to the contigs using BWA 0.7.12-r1039 [11]. Nanopore reads were mapped using the 'bwa mem -x ont2d' option. The resulting SAM files were converted to BAM files, sorted and indexed using samtools 1.2 [12]. Coverage was calculated using samtools depth [Illumina – 49.8(7.1)/333.9(18.3)/7322.9(85.6), Nanopore – 7.4(2.7)/4.5(2.1)/2.9(1.7) : Chromosome/PlasmidA/PlasmidB (SD)].

Results and Discussion

A hybrid assembly using a low coverage of MinION reads (7-8x) combined with moderate coverage Illumina reads (~50x) was used to generate a complete, closed genome. The assembly resolved regions of the genome that were problematic for short read assembly alone, such as chromosomal rRNA operons. The generation of a closed genome from only ~5 % of the possible current yield of a MinION run using a multiplexed library should represent a cost effective means to close multiple genomes during a single MINion sequencing run, although the approach also requires matching short-read Illumina data. Larger or more complex bacterial genomes may require higher coverage read data alongside additional bioinformatics analyses to generate comparably polished, closed genomes [3].

The chromosome showed minor differences to the USA300 reference genome USA300_FPR3757 including 163 SNP differences and the loss and gain of mobile genetic elements (Figure 2). There was minor sequence dissimilarity, including a small deletion, in two of the ribosomal RNA operons . This could either reflect evolutionary changes in these highly conserved sequences or minor misassembly; these regions are typically difficult to assemble. MHO_001 lacked Staphylococcal pathogenicity island 5 (SAPI5), a 13,960 bp exotoxin encoding transposon observed at position

881,852 in the reference. MHO_001 also lacked the prophage phiSA3USA which harbours the important virulence factor staphylokinase. As the integration site of this phage (the *hlb* gene) is intact it is possible that MHO_001 has never acquired this phage. MHO_001 contained a 42,297-bp tyrosine recombinase bacteriophage integrated at position 867,385. This bacteriophage was contained a beta-lactamase and a putative Panton-Valentine-like leuckocidin and several hypothetical genes. The position of an insertion sequence containing ftsK translocase differs between MHO_001 and the reference genome, consistent with a translocation event (USA300_FPR3757:1630720-1644076 to MHO_001:679522-692877). The location of this element in MHO_001 truncates a gene of unknown function. There is a short 1282 bp deletion of a gene encoding an exotoxin at position 448,767 in MHO_001. MHO_001 also has an extended tRNA cluster at 554,826 containing 7 additional tRNAs (val, thr, lys, gly, leu, arg, pro) relative to USA300_FPR3757, representing either gene expansion, or reduction of this gene cluster in USA300_FPR3757.

A BLAST search revealed that the two smaller contigs were identical to previously sequenced plasmids associated with USA300 [13]. The larger of the plasmids contained an N-type replication system (repA) with a pSK1 type plasmid partitioning system. It encoded a host of resistance genotypes including macrolide (mac), erythromycin (ery), cadmium (cadX and cadD), streptothricin (sta), aminoglycoside (aad), neomycin and kanamycin (aph) resistance genes. In addition to this the plasmid contained a Tn552-like transposon containing a beta-lactam resistance (bin, blaI, blaR1, blaZ) operon and a sin recombinase. The smaller of the two plasmids encoded three hypothetical proteins and a replicase. Both plasmids have been previously observed to occur concurrently in the same host.

There was a discrepancy observed between the coverage of short and long reads of plasmidic and chromosomal contigs (Figure 2). The short read coverage of plasmids A and B was on average 6.7x and 149x higher, respectively, than the coverage of the chromosome. The opposite trend was observed with long reads; the plasmids had 0.6x and 0.4x coverage compared to the chromosome. In addition to this the smaller of the two plasmids was only intermittently covered by MinION reads.

The reduced number of mappable nanopore reads was likely due to the fragment size selection steps during library preparation. The inherent problems of aligning long error-prone reads to reference sequences may also have contributed. It is thus important that future studies attempting to reconstruct plasmids or studying plasmid diversity consider the impact of size selection on downstream analysis or to prepare multiple DNA libraries with differential size selection as previously discussed by Koren and Phillippy [14]. However, the clear benefit of hybrid sequencing is that it allows for the identification and correct assembly of features that would be otherwise lost using one method sequencing method preferentially over the other.

Competing interests

No competing interests.

Funding

The authors would like to acknowledge BBSRC/NERC grant number BB/M026388/1 for providing funding for SB. SB and VH were also funded by a grant from the United Kingdom Clinical Research Collaboration (UKCRC) Translational Infection Research (TIR) initiative, and the Medical Research Council (Grant Number G1000803, held by Prof. Sharon Peacock) with contributions from the Biotechnology and Biological Sciences Research Council, the National Institute for Health Research on behalf of the Department of Health, and the Chief Scientist Office of the Scottish Government Health Directorate. The authors are grateful for travel funds provided by NERC (NE/N000501/1) for SB and Medical Research Council Cloud Infrastructure for Microbial Bioinformatics (CLIMB) for VH to attend.

Authors Contributions

SB and VH were responsible for the conception and design of study and data acquisition. SB

performed the analysis and interpretation of data and manuscript drafting. HF and EF revised the manuscript critically for important intellectual content. SB and EF approved the version of the manuscript to be published.

Acknowledgements

The authors would like to acknowledge the contribution of Nick Loman and the rest of the organizing committee of the PoreCamp 2015 nanopore training workshop which was hosted in the Centre for Computational Biology at the University of Birmingham in December 2015. We would like to thank Oxford Nanopore for allowing Bath University access to the MinION Access Programme (MAP). The authors would also like to acknowledge Maho Yokoyama for her contributions and technical assistance.

Data Availability

The dataset supporting the conclusions of this article is available in the European Nucleotide Archive repository under project number PRJEB14152.

References

1. Glaser P, Martins-Simões P, Villain A, Barbier M, Tristan A, Bouchier C, et al. Demography and Intercontinental Spread of the USA300 Community-Acquired Methicillin-Resistant Staphylococcus aureus Lineage. MBio. 2016;7:e02183–15.

2. Loman NJ, Quick J, Simpson JT. A complete bacterial genome assembled de novo using only nanopore sequencing data. Nat. Methods. 2015;12:733–5.

3. Risse J, Thomson M, Patrick S, Blakely G, Koutsovoulos G, Blaxter M, et al. A single chromosome assembly of Bacteroides fragilis strain BE1 from Illumina and MinION nanopore sequencing data. Gigascience. 2015;4:60.

4. Karlsson E, Lärkeryd A, Sjödin A, Forsman M, Stenberg P. Scaffolding of a bacterial genome using MinION nanopore sequencing. Sci. Rep. 2015;5:11996.

5. Madoui M-A, Engelen S, Cruaud C, Belser C, Bertrand L, Alberti A, et al. Genome assembly using Nanopore-guided long and error-free DNA reads. BMC Genomics. 2015;16:327.

6. Bolger AM, Lohse M, Usadel B. Trimmomatic: a flexible trimmer for Illumina sequence data. Bioinformatics. 2014;30:2114–20.

7. Bankevich A, Nurk S, Antipov D, Gurevich AA, Dvorkin M, Kulikov AS, et al. SPAdes: a new genome assembly algorithm and its applications to single-cell sequencing. J. Comput. Biol. 2012;19:455–77.

8. Diep BA, Gill SR, Chang RF, Phan TH, Chen JH, Davidson MG, et al. Complete genome sequence of USA300, an epidemic clone of community-acquired meticillin-resistant Staphylococcus aureus. Lancet. 2006;367:731–9.

9. Darling AE, Mau B, Perna NT. progressiveMauve: multiple genome alignment with gene gain, loss and rearrangement. PLoS One. 2010;5:e11147.

10. Seemann T. Prokka: rapid prokaryotic genome annotation. Bioinformatics. 2014;30:2068–9.

11. Li H, Durbin R. Fast and accurate short read alignment with Burrows-Wheeler transform. Bioinformatics. 2009;25:1754–60.

12. Li H, Handsaker B, Wysoker A, Fennell T, Ruan J, Homer N, et al. The Sequence Alignment/Map format and SAMtools. Bioinformatics. 2009;25:2078–9.

13. Shearer JES, Wireman J, Hostetler J, Forberger H, Borman J, Gill J, et al. Major families of multiresistant plasmids from geographically and epidemiologically diverse staphylococci. G3. 2011;1:581–91.

14. Koren S, Phillippy AM. One chromosome, one contig: Complete microbial genomes from long-read sequencing and assembly. Curr. Opin. Microbiol. 2015. p. 110–20.

Figures

Fig 1. Read length histogram of nanopore reads. 2D reads with a phred score greater than 8 were classified by Metrichore as pass reads (blue), all other 2D reads were classified as fail reads (blue). The bin width was set to 500 bp.

Fig 2. Alignment of MHO 001 chromosome and plasmids to reference assemblies with corresponding long and short read coverage. The backbone sequence alignments between MH0 001 and reference sequences were identified using MAUVE [9] and displayed using red blocks. Inversions are displayed in blue. Open reading frames are annotated as blue blocks with the exception of rRNA which is represented by red blocks. Those above the line representing the contig are on the forward strand and those under the line are on the reverse strand. Notable mobile genetic elements or genomic features are annotated. A scale bar in bp is present underneath each sequence. Per base read coverage of MinION long reads (blue) and Illumina short reads (orange) is shown above each sequence. The coverage y-axis has been constrained to 20 reads for MinION reads. The coverage y-axis has been constrained to 200, 350 and 8000 reads for short read coverage of MHO 001, Plasmid A and Plasmid B respectively. The number of bins was set to 1000 for each sequence.

Figure 1

Click here to download Figure Figure1.pdf

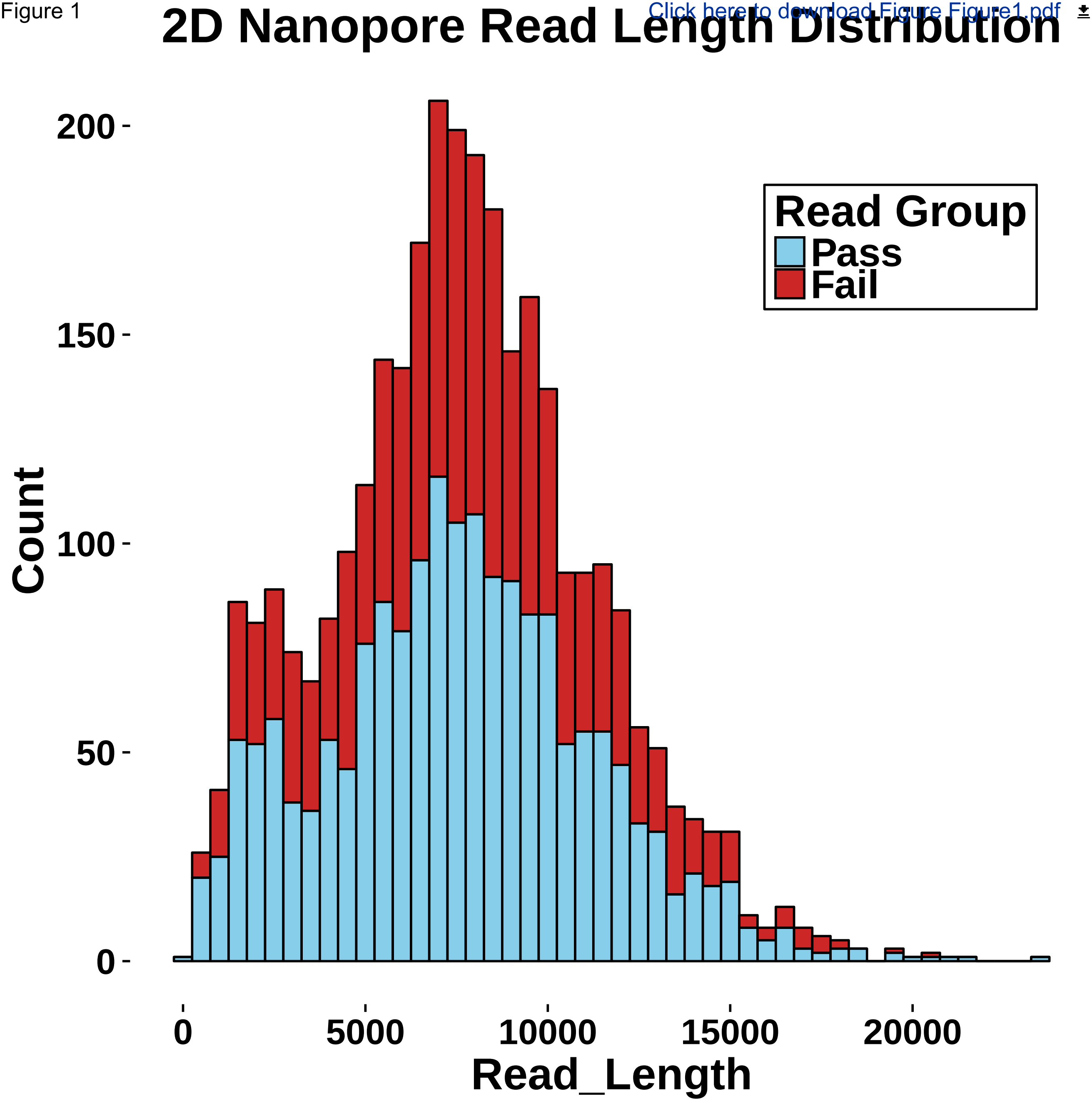

Figure 2

Click here to download Figure Figure2.pdf

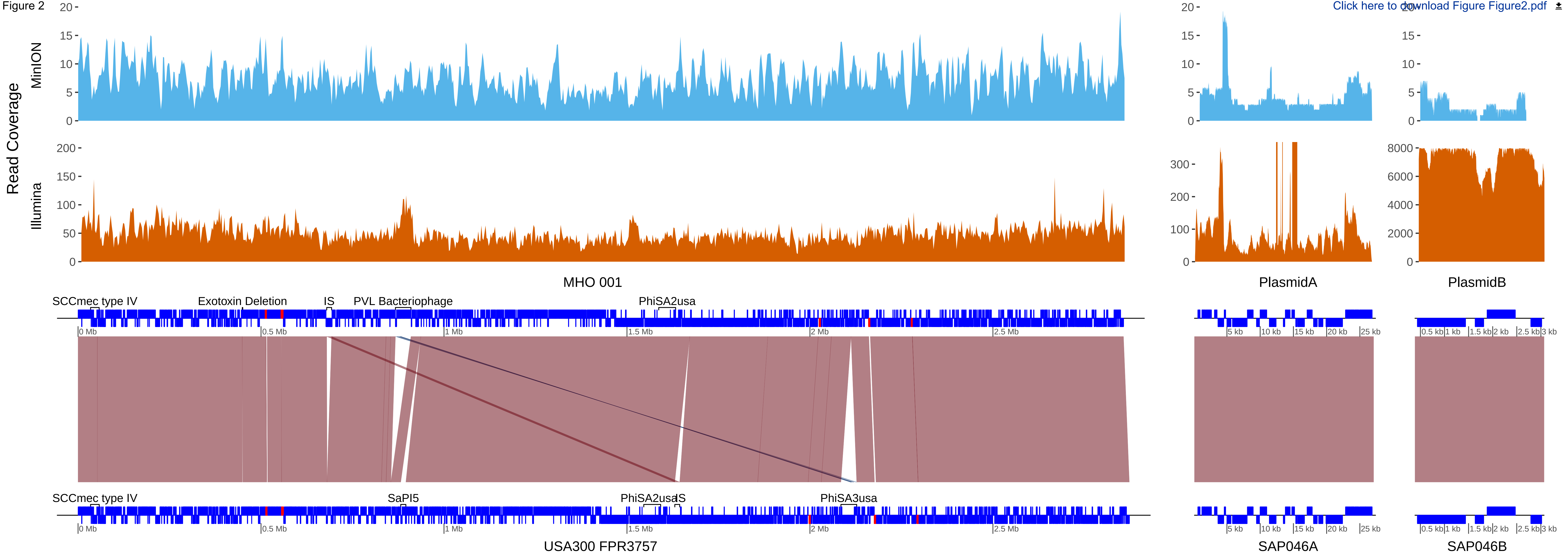

Click here to access/download
Supplementary Material
Readme.txt

Data Description

Click here to access/download
Supplementary Material
DataDescription.docx
