# Supplementary material for: The use of Oxford Nanopore native barcoding for complete genome assembly
Source: Gigascience. 2017 Feb 24;6(3):1–6. doi: 10.1093/gigascience/gix001 (PMC5467021; doi:10.1093/gigascience/gix001)
Supplement: Supplement Files [file gix001_supp.zip › Supp_Table1.pdf]

	Filtered	Unbarcoded
# Reads	9774	9501
# BLASR Hits (% # Reads)	28 (0.29%)	722 (7.60%)
Mean Alignment Length (%)	45.61	81.22
Mean Percentage Similarity (%)	75.27	75.07
# Hits < 75% Read Length (%)	16 (57.14%)	153 (21.19%)
# Hits >= 75% Read Length (%)	12 (42.86%)	569 (78.81%)
