# Supplementary material for: The use of Oxford Nanopore native barcoding for complete genome assembly
Source: Gigascience. 2017 Feb 24;6(3):1–6. doi: 10.1093/gigascience/gix001 (PMC5467021; doi:10.1093/gigascience/gix001)
Supplement: Supplement Files [file gix001_supp.zip › Supplementary_Analysis.pdf]

Supplementary Analysis

Materials and Method

PCR was used to confirm the presence of structural variants in MHO_001 as inferred from our assembly. The expansion of a tRNA island and absence of SAPI5 were selected as examples. Strain JE2, a USA300 clone strain derived from LAC which is 11 SNPs different from USA300_FPR3757 [1] (the reference genome used in the main text), was used for comparison to MHO_001.

In order to identify the expansion of the tRNA island, primers were designed flanking the region (Supplementary Analysis Table 1; Supplementary Analysis Figure 1). The differential size of the resulting products would identify expansion/truncation (Truncated: 228 bp, Expanded: 878 bp). In order to test for the presence of SAPI5 four primers were designed; two primers flanking SaPI5 and two internal to SaPI5 (Supplementary Analysis Table 1; Supplementary Analysis Figure 1). By using the primers in three pairs, we were able to determine if the island was present or absent in the two strains. The individual primers are numbered in Supplementary Analysis Figure 1 and Supplementary Analysis Table 1. Primer pair A corresponds to primers 1 and 2, pair B corresponds to primers 1 and 4 and pair C corresponds to primers 3 and 4. Pairs A and C amplified regions flanking SaPI5, starting from outside the island and extending towards the centre of SAPI5, which will only amplify if SAPI5 is present in that region of the genome (Product sizes: A - 652 bp, B - 134 bp). Primer Pair B combined the primers of pairs A and C that were external to SAPI5, amplified between these flanking regions. Due to the size of SAPI5, amplification can only occur if the island is absent producing a 117 bp product.

DNA was prepared from JE2 and MHO_001 using the method previously described. PCR was performed using OneTaq Hot Start Master Mix with standard buffer (New England BioLabs) for 30 cycles according to the manufacturer’s guidelines; the annealing temperature was 52.9°C and the extension time was 1 min. The products were mixed with 6x Loading Dye (New England Biolabs) and was run on a 1.5% agarose gel stained with ethidium bromide at 85V alongside a 100bp ladder (New England Biolabs) and visualised using ChemiDoc imager (BioRad) (Supplementary Analysis Figure 2). Samples were purified using a GeneJet PCR Purification kit (Thermo Scientific) and sequenced (Eurofins).

Primer	Pair	Sequence	Tm (°C)
SaPI5_1 F	A and B	CAATGGTGCAGTTATTCCTGC	57.9
SaPI5_2 R	A	CGTATTGGTGAAATGTTAGCCATTC	59.7
SaPI5_3 F	C	GCCCTTGAACTTCCTTGATAATC	58.9
SaPI5_4 R	B and C	GAATAAATGAGTAGCACGCATGC	58.9
tRNA island F	tRNA Island	GCTGATGTGACAAAGTTTACATCAA	58.1
tRNA island R	tRNA Island	CCGCTGACCTCCTGCG	59.4

Supplementary Analysis Table 1. PCR primers used to identify tRNA expansion and presence of SAPI5.

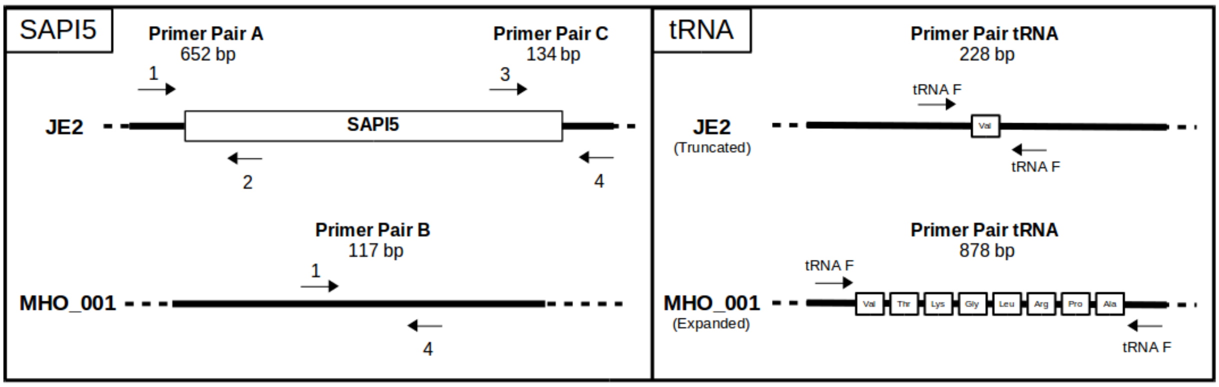

Supplementary Analysis Figure 1. Schematic representation of the PCR primer pairs used to identify the presence or absence of SAPI5 and an expanded tRNA island in MHO_001 and reference strain JE2. The expected product sizes are shown below the primer pairs.

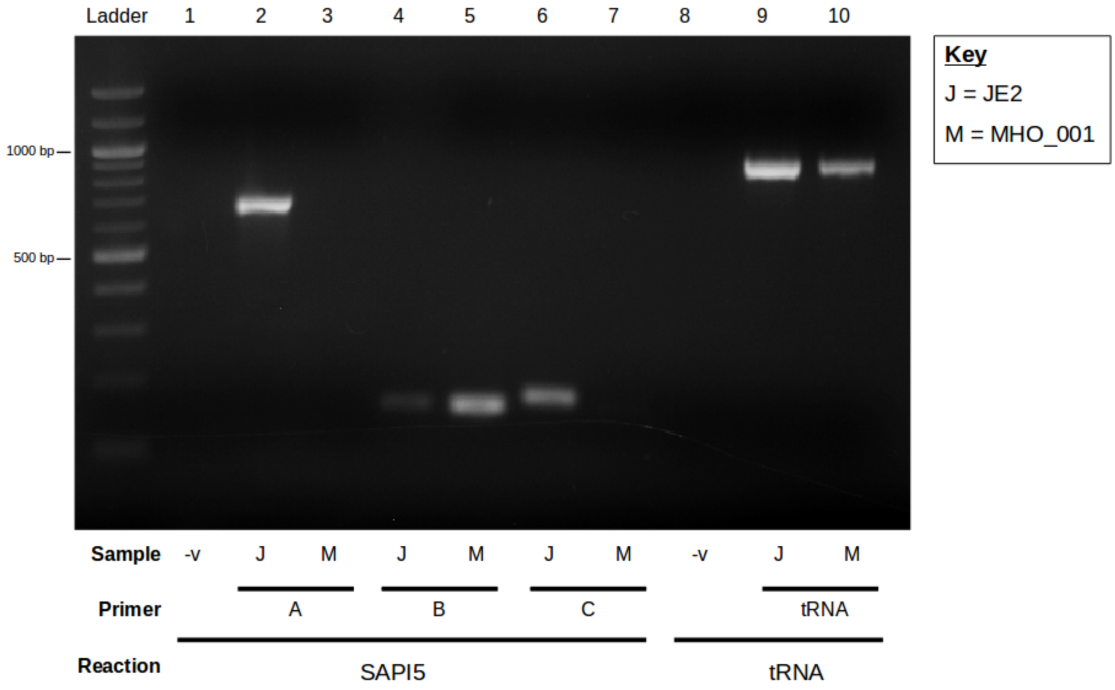

Supplementary Analysis Figure 2. Agarose gel image of the PCR to identify the expansion of tRNA island expansion and SAPI5 presence or absence. 100 bp ladder (NEB) was used for band sizing.

Results and Discussion

The presence of a band at 878 bp in both both MH0_001 and JE2 suggested that tRNA island was present in its longer variant in both genomes. These results support the sequence described in the main manuscript and additionally suggested that either the tRNA island was truncated in the strain used to generate the reference genome or the reference sequence was in error.

In MHO_001 only primer pair B amplified, indicating absence of SAPI5. In JE2, primer pairs A and C amplified, indicting the presence of SAPI5. There was also weak amplification of primer pair B with an amplicon of the same length and sequence as observed in MHO_001, either indicating low levels of non-specific priming or perhaps that SAPI5 is intermittently lost during culture. All amplicons were sequenced, and the sequences confirmed that these products were the predicted genomic regions. The raw sequence data has been archived in the Gigascience repository linked with this publication and also replicated below.

In conclusion, this analysis confirmed the two structural variants in MH01_001 as inferred from our genome assembly. The results for the reference genome were not exactly as predicted from the USA300_FPR3757 reference sequence, but this is likely to reflect the fact that we used USA300 strain JE2, which is a subtle variant of this reference.

References

1. Kennedy AD, Otto M, Braughton KR, Whitney AR, Chen L, Mathema B, et al. Epidemic community-associated methicillin-resistant *Staphylococcus aureus*: recent clonal expansion and diversification. Proc. Natl. Acad. Sci. National Acad Sciences; 2008;105:1327–32.

Amplicon Sequences

>tRNA_F, MHO_001

AGAGGTTAGTACTATTGCACCTTATTATTAAGCGTGTATCATGAATAAGTAAGTTATTTTGTCTGG
TGACTATAGCAAGGAGGTCACACCTGTTCCCATGCCGAACACAGAAGTTAAGCTCCTTAGCGT
CGATGGTAGTCGAACTTACGTTCCGCCAGAGTAGAACGTTGCCAGGCAAATGACAAATCGGAG
AATTAGCTCAGCTGGGAGAGCATCTGCCTTACAAGCAGAGGGTCGGCGGTTCGAACCCGTCA
TTCTCCACCATTTATTCTTAGATATAGCCGGCCTAGCTCAATTGGTAGAGCAACTGACTTGTAAT
CAGTAGGTTGGGGGTTCAAGTCCTCTGGCCGGCACCATCTTTTGAGCCATTAGCTCAGCTGGT
AGAGCATCTGACTTTTAATCAGAGGGTCAGAGGTTCGAATCCTCTATGGCTCATTACGATTTAAT
TTTTATATTTAGCAAAATAATGCAGAAGTAGTTCAGCGGTAGAATACAACCTTGCCAAGGTTGGG
GTCGCGGGTTCGAATCCCGTCTTCTGCTCCATTATTTTGTCGGGGTGGCGGAACTGTGAGACA
CACAGGACTTATAATACAGCGGTGAGAGAAAACGTACCGGTTCGATTCCGGACCCCCGCACTG
TTTTAGCGACCGTCTCTCAACTGAATAGAGTCTTCGACTAGTGATCAGCCTGGTAAGGGTCCGA
TCTCGGCTCAAGCGCGCTCTACTATCTTCAATGAATAGGAGGAAATATTCTCGTGTTGGTATAGC
AAACGGTTGGGACCCACTGGTCTATGCTCGAATCAGTACTTACTATTATCTGAAAATGCTTATTAT
GAGGCGGAGCACATCAGTTTAAGCGCTGACGTTGCCTTGTCACGCGGCAGAA

>tRNA_R, MHO_001

GAGAAACCTCAGCTGAGCTAGCCCCATAAATGGAATTTAAGAAGTAATCGGGAAGACAGGATTC
GAACCTGCGACCCCTTGGTCCCAAACCAAGTGCTCTACCAAGCTGAGCTACTTCCCGTTATTC

AATTAATTTAAAAATGGCGCGCCCGATAGGAGTCGAACCCATAACCTCTTGATCCGTAGTCAAA
CGCTCTATCCAATTGAGCTACGGGCGCTAAAATGGTGCCGAGGACCGGAATCGAACCGGTAC
GGTGATCTCTCACCGCAGGATTTTAAGTCCTGTGCGTCTGCCAGTTCCGCCACCCCGGCAAAA
TAATGGAGCAGAAGACGGGATTCGAACCCGCGACCCCAACCTTGGCAAGGTTGTATTCTACCG
CTGAACTACTTCTGCATTATTTTGCTAAATATAAAAATTAAATCGTAATGAGCCATAGAGGATTCG
AACCTCTGACCCTCTGATTAAAAGTCAGATGCTCTACCAGCTGAGCTAATGGCTCAAAAGATGG
TGCCGGCCAGAGGACTTGAACCCCCAACCTACTGATTACAAGTCAGTTGCTCTACCAATTGAG
CTAGGCCGGCTATATCTAAGAATAAATGGTGGAGAATGACGGGTTCGAACCGCCGACCCTCTG
CTTGTAAGGCAGATGCTCTCCCAGCTGAGCTAATTCTCCGATTTGTCATTTGCCTGGCAACGTT
CTACTCTGGCGGAACGTAAGTTCGACTACCATCGACGCTAAGGAGCTTAACTTCTGTGTTCGG
CATGGGAACAGGTGTGACCTCCTTGCTATAGTCACCAGACAAAATAACTTACTTATTCATGATAC
ACGCTTAATTAATAAAGTGCAATAGTTAATTTTACACTTTGATGTAACTGTGCTCAATACTCATACA
GCAAA

>tRNA_F, JE2

TCACGTAGTACTATTGCACCTTATTATTAAGCGTGTATCATGAATAAGTAAGTTATTTTGTCTGGT
GACTATAGCAAGGAGGTCACACCTGTTCCCATGCCGAACACAGAAGTTAAGCTCCTTAGCGTC
GATGGTAGTCGAACTTACGTTCCGCCAGAGTAGAACGTTGCCAGGCAAATGACAAATCGGAGA
ATTAGCTCAGCTGGGAGAGCATCTGCCTTACAAGCAGAGGGTCGGCGGTTCGAACCCGTCAT
TCTCCACCATTTATTCTTAGATATAGCCGGCCTAGCTCAATTGGTAGAGCAACTGACTTGTAATC
AGTAGGTTGGGGGTTCAAGTCCTCTGGCCGGCACCATCTTTTGAGCCATTAGCTCAGCTGGTA
GAGCATCTGACTTTTAATCAGAGGGTCAGAGGTTCGAATCCTCTATGGCTCATTACGATTTAATT
TTTATATTTAGCAAAATAATGCAGAAGTAGTTCAGCGGTAGAATACAACCTTGCCAAGGTTGGGG
TCGCGGGTTCGAATCCCGTCTTCTGCTCCATTATTTTGTCGGGGTGGCGGAACTGTCAGACAC
ACAGGACTTAAAATACTGCGGTGGGAGATCACCGTCCCGGTTCGATTCCCGACCCCCGCCCC
ATAATAGCGACCGTCTCTCAACTGAATAGATAGTTCGACTACGGATCAGGAGGTATGGGTTCAA
CCCGGAGCAAGCGAGCTTCTATTACATTAATGATTAAGAGGATGTATCTATGTTGGTGAGGAAAC
AGATTGGACTCCACTGCGTCGATGCTCGACCAAGTACTCTCGATATACTGTACATCCATTTTAAG
AAGGTGCAGCTACATCTGTTAAGCCTCTGCGTTGACCTTCGTGACGTCCGCAGAAC

>tRNA_R, JE2

ATGACTTCAGCTGAGCTAGCCCCCATAAATGGAATTTAAGAAGTAATCGGGAAGACAGGATTCG
AACCTGCGACCCCTTGGTCCCAAACCAAGTGCTCTACCAAGCTGAGCTACTTCCCGTTATTCA
ATTAATTTAAAAATGGCGCGCCCGATAGGAGTCGAACCCATAACCTCTTGATCCGTAGTCAAAC
GCTCTATCCAATTGAGCTACGGGCGCTAAAATGGTGCCGAGGACCGGAATCGAACCGGTACG
GTGATCTCTCACCGCAGGATTTTAAGTCCTGTGCGTCTGCCAGTTCCGCCACCCCGGCAAAAT
AATGGAGCAGAAGACGGGATTCGAACCCGCGACCCCAACCTTGGCAAGGTTGTATTCTACCG
CTGAACTACTTCTGCATTATTTTGCTAAATATAAAAATTAAATCGTAATGAGCCATAGAGGATTCG
AACCTCTGACCCTCTGATTAAAAGTCAGATGCTCTACCAGCTGAGCTAATGGCTCAAAAGATGG
TGCCGGCCAGAGGACTTGAACCCCCAACCTACTGATTACAAGTCAGTTGCTCTACCAATTGAG
CTAGGCCGGCTATATCTAAGAATAAATGGTGGAGAATGACGGGTTCGAACCGCCGACCCTCTG
CTTGTAAGGCAGATGCTCTCCCAGCTGAGCTAATTCTCCGATTTGTCATTTGCCTGGCAACGTT
CTACTCTGGCGGAACGTAAGTTCGACTACCATCGACGCTAAGGAGCTTAACTTCTGTGTTCGG
CATGGGAACAGGTGTGACCTCCTTGCTATAGTCACCAGACAAAATAACTTACTTATTCATGATAC
ACGCTTAATTAATAAGGTGCAATAGTTAATTTTACACTTTGATTACGTTTTTTTTTAAAAAAAAAAA
AAATGTTACGGGAGCCTACC

>Primer Pair A, SAPI5_1, JE2

TTAAGATCTTTGTTATAAATACCCTTTATAACAACGATTAAGGGGTTATTTTTGTGGTATTGAAAAT
AAAAAAGGGCACAAAAGGGGCAGTATATGCAAATTATCGAATTTTAAACAAAAAAATACGCTCAG
ATTTGAGCGCATTATCCAAGTTTTACCTGTTCAAGTTTGTTCATCATATCCTTATCCATCTGTTCA
GTAACGTGAGAATAAATGGAAAGTGTTGTACGGTGGTCGGAATGGCCTACACGATCCATAATAG
CTTTAAGTGACACGCCTTGTTGAGAGAGTAATGATATGTGGCTATGTCTTAATATATGTGAAGAA
ACTTCTTTATCAATACCAACATCTTTAGCTGCTTCCCTAAGGATTTTATTGAATCTTTCAGTCTGC
ATTGGGTTGCCTTTATGATTAGTAAATACAAAATTTCTATTTAGATATCCATCATTCCATTTTGAATC
CTTTTTGTTCTCCAGTATTGCTTTCTTTAATATCTCGCAACTTCTGCTACTCAATCCAATTGTTCTA
TAACTAGACTCTGTTTTAGTGGTATCTTTTACACCGAATCCACCAGATTCATCGTGAAACCAATG
GATTGTTCCGTTAATATTTAAACTCTTATTATCAAAGTCTATATCTTCATTTTGAATGGCTAACATCA
CCCCCATATCCGAA

>Primer Pair A, SAPI5_2, JE2

GGTAACAAGAATCATAGACTGTGGAAGCGCAGCACGATTAAATATTAACGGAACAATCCATTGG
TTTCACGATGAATCTGGTGGATTCGGTGTAAAAGATACCACTAAAACAGAGTCTAGTTATAGAAC
AATTGGATTGAGTAGCAGAAGTTGCGAGATATTAAAGAAAGCAATACTGGAGAACAAAAAGGAT
TCAAAATGGAATGATGGATATCTAAATAGAAATTTTGTATTTACTAATCATAAAGGCAACCCAATG
CAGACTGAAAGATTCAATAAAATCCTTAGGGAAGCAGCTAAAGATGTTGGTATTGATAAAGAAGT
TTCTTCACATATATTAAGACATAGCCACATATCATTACTCTCTCAACAAGGCGTGTCACTTAAAGC
TATTATGGATCGTGTAGGCCATTCCGACCACCGTACAACACTTTCCATTTATTCTCACGTTACTG
AACAGATGGATAAGGATATGATGAACAAACTTGAACAGGTAAAACTTGGATAATGCGCTCAAATC
TGAGCGTATTTTTTTGTTTAAAATTCGATAATTTGCATATACTGCCCCTTTTGTGCCCTTTTTTATT
TTCAATACCACAAAAATAACCCCTTAATCGTTGTTATTAAAGGGTATTTTTATTTAATTGGTATTATT
TAGCAGGAATTGTCGGCCCCATTGTGAAAGGGGGCTTTATTTCTTGGAAGACGATCGCTGGGT
GCTAATATACACACGAGCGCTCGCCAGCTCGGAAGTATCAGAAGATCCGACTCTCTTGACCCC
CGCCGCAGGCACACAAGAAGACGATACGAGACAGACACGACGCCTGCGACATACAGCTAGCG
TTGGGTATGCTGTTGGTGAGGTATGAGAGATCCCACGTCGTTGCAGAGGCTGGCAGTTGGGA
ACACCATTGACACTCTGCTCAAAACTATAGAGAGAAAGAGTGCTGCTGCACACAAAACGAAGT
CAAATTTGCTTCCGGAGATCCCGACCATTGATAGTCGCATCCACTGCGTATTCCCGTCCAACCA
CCCTTAGTCGTAGACGTTCA

>Primer Pair C, SAPI5_3, JE2

AAGTCATCTGCTATAATCATATTAATGGCTCAGTAACGTGATGTTGCTGGGCTTTTTAATTTAAAC
AGGTATTTATATGATATTTAGGAATGAGATGCTATTACGGATAAAGTAAAATCCTATATGCATGCGT
GCTACTCATTTATTCTGACGTGTTCAAGTTTGTTCATCATATCCTCATCCATCTGTTCGCTACCGA
GATTCTAAAATGGAAAGTGATGTACGACGGCCGGTACGGCCTACACCATCCCTAAGAGCTTTAC
CTGCCACGCCTTGATGAGACAGTAATGATATGTGGCTATGTCTTAATAAATGTGAAGATTATTCTT
TATCTCACCAACATCTGTATCTGCCTCCCTAAGGATTTTATTGAACCTTTTGCTCTGCACTGGGA

>Primer Pair C, SAPI5_4, JE2

TAGTCATCGGAATCTTTATCGTATAGCATCTCATTCCTAAATATCATATAAATACCTGTTTAAATTAA
AAAGCCCAGCAACATCACGTTACTGAGCCATTAATATGATTTATTTAGCAGGAATAATTAGCCAGA
TTATCAAGGAAGTTCAAGGGC

>Primer Pair B, SAPI5_1, MHO_001

TTTAAAATTTTTGGGTATGGCTCAGTACGTGATGTTGCTGGGCTTTTTAATTTAAACAGGTATTTA
TATGATATTTAGGAATGAGATGCTATTACGGATAAAGTAAAATCCTATATGCATGCGTGCTACTCAT
TTATTCAGGTTAAGTGCCTCAATGACTAGTGAGTCCATAACTTCTATTTACATATCCCCATTCCCT
TTTGACTCCTCCTTGTTTCCTATAT

>Primer Pair B, SAPI5_4, MHO_001

TACTTCGTCGATTTCTGTATCGTATAGCATCTCATTCCTAAATATCATATAAATACCTGTTTAAATTA
GAAAGCCCAGCAACATCACGTTACTGAGCCATTAATATGATTTATTTACCAGGAAGAACTGCACC
ATTGCATACTTCTCTGCCTGCCTGGAACTAGGTAAGTATTCCCTTCTAGACGCCCGAACAGGAT
AGGACGAAGGTCTATTACTCCACTCATGTATATTTCTTATATTTTTATCTGGTTTCAATATAGATAT
GCTTGACATACTGGCCCAAAACTTTTTTGATTTGTTCTGATACCAAAAAAAGAACATGCTAAGTG
GGGAGAGCATTCACCCGTCCATCA

>Primer Pair B, SAPI5_1, JE2

TTGAATTTATTGTATATGGCTCAGTACGTGATGTTGCTGGGCTTTTTAATTTAAACAGGTATTTATA
TGATATTTAGGAATGAGATGCTATTACGGATAAAGTAAAATCCTATATGCATGCGTGCTACTCATTT
ATTCAGGCCATACTGTTGCCCACCGGGGTATCAATTGGTACAACTTGCGGCCGAGACCGATCG
CGGGCCTGACTTAAATGGTTTCGTAACGAACAATCACGGCGACTACCATACCCTTTCTGAGAG
GGTGACCATCCTTCCCGCGACGGGACACGGTCCCAAAACTCCTTGCTCCGGTATTTAGGTGG
AAAATTGAATTTAGCACGCAATTTGGACGG

>Primer Pair B, SAPI5_4, JE2

TGTCTCGCATACTGTATCGTATAGCGTCGCATGTCCGTGACTATCATATTTATACCTGTATTATATT
AGAAAGCCCAGCATCGTCACGCTTACTGAGCCATTAATATGACTTATTTGCCAGGAAGAACTGC
ACCATTGCATACTGCATTTCTGGCACAAGGTATGTTACCATTTTAATATGCGTTGATATGGTAACA
GGAGGCTCGAAAAAAGCCACTCAACAAATGACATCAACTAATACTTTTCTCAGCTCCCCTAATTA

AATCTGCCGCAGCGAGTGGGCCAAAACTCTTCTTTTTTGTTAACACCAAAAATACCGCTTCTGA
TTCAGTAGGAGTGGGCCTTCACCCACCTGTCG
