# Supplementary figures and images for: The use of Oxford Nanopore native barcoding for complete genome assembly
Source: Gigascience. 2017 Feb 24;6(3):1–6. doi: 10.1093/gigascience/gix001 (PMC5467021; doi:10.1093/gigascience/gix001)

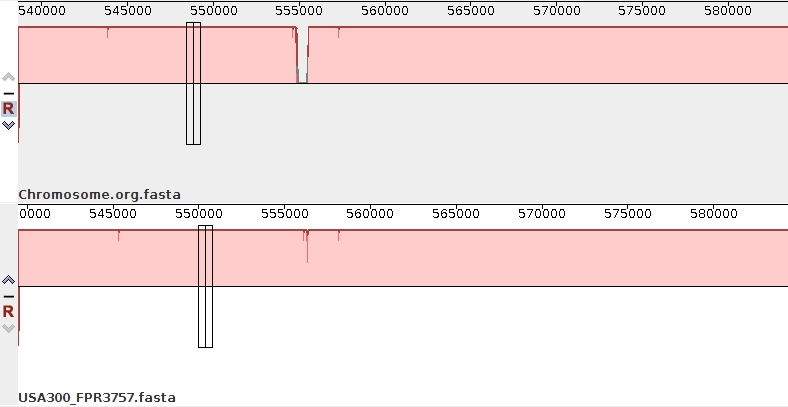

Supplement: Supplement Files [file gix001_supp.zip › Supp_Figure1.jpg]

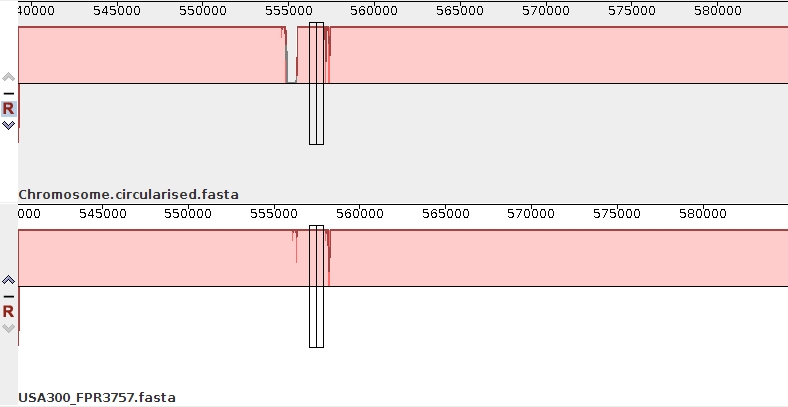

Supplement: Supplement Files [file gix001_supp.zip › Supp_Figure2.jpg]

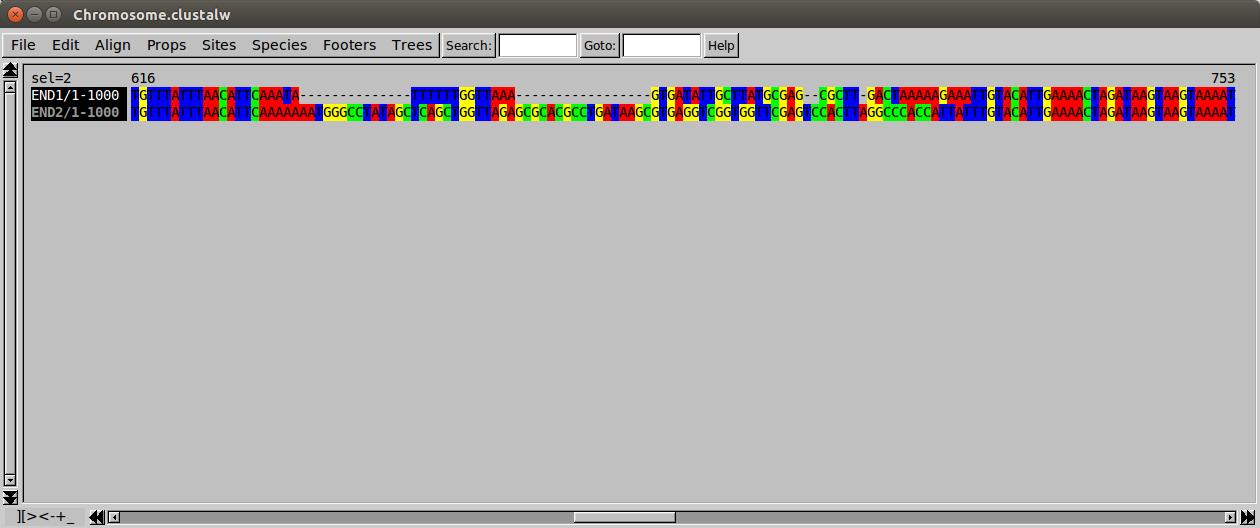

Supplement: Supplement Files [file gix001_supp.zip › Supp_Figure3.jpg]

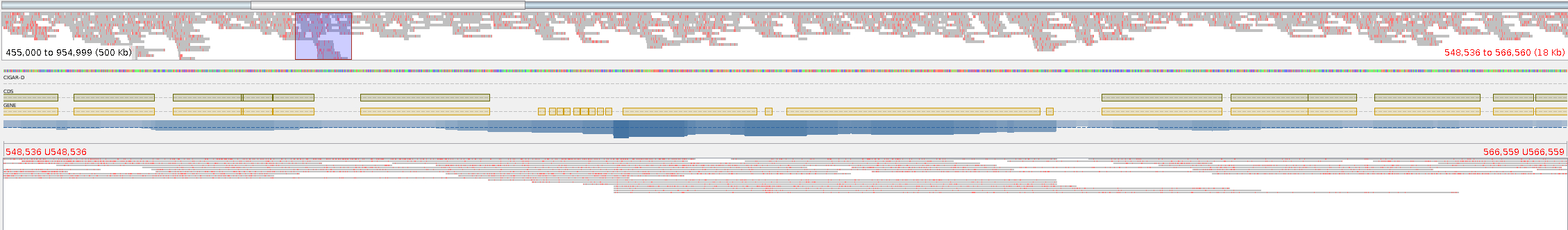

Supplement: Supplement Files [file gix001_supp.zip › Supp_Figure4.jpeg]
