# Supplementary material for: The use of Oxford Nanopore native barcoding for complete genome assembly
Source: Gigascience. 2017 Feb 24;6(3):1–6. doi: 10.1093/gigascience/gix001 (PMC5467021; doi:10.1093/gigascience/gix001)
Supplement: Response_to_reviewers_Orginal_Submission.pdf [file gix001_response_to_reviewers_orginal_submission.pdf]

Response to reviewers:

We would first like to thank the reviewers for taking time to provide detailed and insightful responses to the manuscript. We are confident that we have addressed these legitimate concerns with significant modifications to the manuscript and additional analysis. We hope that you share our view that the manuscript is now substantially improved, and provides more convincing support for the conclusions drawn. Moreover, we feel that the additional technical analysis widens the appeal of the paper by making the workflow more accessible.

Revisions

Reviewer #1 (JM Aury)

The manuscript "The use of Oxford Nanopore native barcoding for complete genome assembly" by Bayliss et al. describes a methodology dedicated to genome assembly of small genomes by combining the MinION device with the Illumina sequencing technology. Several studies have already reported the use of the Oxford Nanopore technology for genome assembly, as mentioned by the authors. Here, authors take advantage of the possibility of multiplexing libraries on a single sequencing run. The manuscript describes the sequencing data and the minor differences between the USA300 reference genome and the MHO_001 strain. The assembly method is classical and has already been used in Risse et al. (GigaScience 2015) to assemble the Bacteroides fragilis genome. My main concern about the manuscript is related to the weak description and analysis of the multiplexed data. In addition, the impact is limited because the method used and the results obtained improve slightly the state of the art for nanopore-based assembly.

Major Compulsory Revisions

1) I recommend the authors to give a better insight of the usage of barcodes. Several questions are emerging, like for example the accuracy of the demultiplexing process (given the high error rate of nanopore reads), the fraction of cross-contamination (reads that are assigned to a wrong sample). These two informations could affect the required sequencing coverage.

We thank the reviewer for raising this important point. In response to this and point 4) below we evaluated how the reads were demultiplexed (revising the demultiplexing script in the process, see point 4 below) and aligned the reads to the complete genome produced by the assembly using the BLASR long read aligner. The revised filtering script (FilterBarcodes.pl) allowed for more stringent parameters to be used for identifying barcodes in the 2D failed reads. This produced a set of 1499 2D failed reads. The failed 2D reads were combined with the 1324 2D pass reads and the Illumina reads to produce an identical assembly to that used during the previous workflow (which used a slightly larger set of nanopore reads, 3105 vs 2821). The pass and fail reads were then aligned to this assembly using BLASR. This resulted in 99.70% of the pass reads and 86.19% of the failed reads aligning to the reference genome. The aligned pass reads had an average 85.87% sequence identity to the reference genome. The aligned fail reads had an average 77.76 % sequence identity to the reference genome. We also ran BLASR on the filtered reads that contained barcodes for other, non-target multiplexed samples and the 2D fail reads in which I was unable to detect a barcode. Only a low percentage of these reads aligned to the reference genome, 27/9774 (0.29%) of barcoded non-sample and 722/9501(7.60%) of unbarcoded reads. In summary, only ~15% of the demultiplexed reads represented either contamination during library prep or a failure to correctly identify the barcode. The majority of the pass reads were correctly demultiplexed by Metrichor.
These methodologies and results have been added to the manuscript (Lines 129 and 154), Figure 1 has been undated accordingly and Table 1 has been added to summarise the BLASR results.

2) The samples used for the pooled library are not defined. Authors should add a full description of the dataset. I couldn't find it at the given URL: http://porecamp.github.io

We apologise for the lack of clarity here. The other isolates were provided by attendees of the Porecamp workshop and these data are being prepared for a separate publication. Inclusion of the very diverse samples from such a broad assemblage of organisms (including eukaryotes) and isolation methodologies would be best served as a separate manuscript. The purview of the current manuscript is focused on the problem of hybrid assembly for bacterial genomes. We have provided additional analysis of the demultiplexed reads (both pass and fail) and confirmation of SNPs by short read variant calling (see Reviewer 2 Point 1 below), and are confident we have addressed these problems and clarified the methodology. We have modified the text to read “The additional DNA samples included in the pooled library were a diverse assemblage of bacterial and eukaryotic DNA samples provided by attendees during the PoreCamp Workshop 2015 at the University of Birmingham. The additional pooled library samples are being prepared for separate publication. Details on the PoreCamp Workshop and associated publications can be found at http://porecamp.github.io/“.

3) Please describe in more details the illumina data. Authors argue that the sequencing was performed on MiSeq and HiSeq platforms, which dataset was used to perform the genome assembly with Spades? What is the read length?

The MiSeq and HiSeq datasets were pooled. The pooled sample represented a sample with sufficient coverage for our analysis. The text now reads “A single 250 bp paired end library was constructed and sequenced on both MiSeq and HiSeq Illumina platforms. The reads from both sequencing runs were combined before downstream analysis”.

4) Please describe in more details how splitbarcodes.py performs the demultiplexing step.

We have modified the text as follows: “2D reads that failed the sample QC were demultiplexed using an in-house script (FilterBarcodes.pl). The twelve 40 bp barcodes used for library construction were compared in a moving 40 bp window to the sequence in the first and last 150 bp of each read. The barcode requiring the least insertions, deletions or substitutions to be permuted into a sequence in the beginning or end of a read, with a maximum cut-off of 14 permutations, was considered a match. Each read could only be assigned to one individual sample, in the case of a tie the reads were discarded. Sequence preceding or following the presence of a barcode at the beginning or end or a read, respectively, were trimmed as adapter sequence”.

Minor Essential Revisions

1) Line 96: Metrichore instead of Metrichor

Corrected in text.

2) Line 104: "...BLAST similarity with with previously..."

Corrected to “The two smaller contigs were 100% identical in both aligned sequence and alignment length to previously sequenced S. aureus lineage USA300 plasmids”

3) Line 165: "...lost using one method sequencing method preferentially..."

The line was revised in response to Reviewer 2 comment 7. The text now reads "However, the clear benefit of hybrid sequencing is that it allows for the generation of larger assemblies with less uncertainties than by using a single sequencing technology preferentially over another".

4) Line 100: Is Spades output a single contig with the same circularization point as the reference genome?

Additional details have been added to the Materials and Methods section: "The contigs were circularised by MUSCLE alignment (default parameters) of identical overlapping regions at the end of contigs and removal of one alternative overlapping sequence using an in-house script (CirculariseOnOverlaps.pl) [8]☐. Start sites were fixed relative to the beginning of the relevant reference sequence". The output of SPAdes v3.6.1 typically produces a redundant overlap of length x at the end of the contig (x often being the length of the K-mer used to produce the contig).

Was this region correctly assembled?

Despite minor discrepancies we are, in general, highly confident in the assembly. The overlapping region in both plasmids was identical. There are a few mismatches between the overlapping regions in the chromosome. This region lies between a 16S and 23s ribosomal RNA which are notoriously hard to assemble correctly. The sequence presented in the manuscript represents the least differences from the USA300 reference genome and is therefore likely to be closer to the 'true' sequence than the alternative. The intergenic regions in all of the ribosomal RNA operons in MHO_001 show some deviation from the USA300_FPR3757 reference. MAUVE alignments/screenshots of the overlap regions compared to USA300_FPR3757 is available as Supplementary Figures 1 and 2. A CLUSTAL alignment of the region is available as Supplementary Figure 3. The pileup file showing the read coverage of long reads in the region and the genome is shown in Supplementary Figure 4. The data used to generate them in available in the GitHub directory. Text has been added to the results - "There was minor sequence dissimilarity, including a small deletion, in ribosomal RNA operons. This could either reflect evolutionary changes in these highly conserved sequences or minor misassembly; these regions are typically difficult to assemble". A clustalw alignment of the overlapping regions is available on the GitHub repository.

Reviewer #2 (AB Paez)

The authors report the complete genome assembly of the Staphylococcus aureus MHO_001 strain using hybrid approaches based on analysis of Illumina and MinION data. Although such type of analysis can be found regularly in the scientific literature, the authors put special attention to the usage of a very small subset of MinION reads to get a complete genome assembly of their pathogenic isolate. MinION data was combined with a the large amount of Illumina reads, obtained by massive and parallel sequencing in MiSeq and HiSeq platforms, to produce three major scaffolds representing the bacterial chromosome of S. aureus MHO_001 and two mobile elements consisting of two plasmids SAP046A.
In my opinion, this work represent an interesting workflow to help in the achievement of valuable information regarding the complete sequencing of bacterial genomes, where the genome structure complexity and the limitations in terms of read length of the second generation of sequencing technologies constitute the major issues. As a consequence, I recommend this manuscript for publication in GigaScience journal once the following minor and major changes have been incorporated to the main text and supporting material:

Major points

1) MinION reads are characterized by their high rate of errors that can reach up to 40% in 1D reads. The

authors declare usage of only 2D reads for the hybrid genome assembly, however, even in this type of reads the per-base accuracy reaches 85% as the most (for R7.3 chemistry). With the above information and the low number of MinION reads used for the manuscript general aim, I would suggest to perform some additional analysis in order to validate the strategy presented. This will imply to corroborate by Sanger sequencing, when technically possible, the genetic variants and chromosome rearrangements found in the MHO_001 strain in comparison with the USA300.

In order to address this point we would like to first comment briefly on the hybrid assembly methodology used in this manuscript. SPAdes does not directly use the error-prone long reads to assemble the resulting contigs. Rather it relies on a methodology called 'read threading' to reduce the complexity of the De Bruijn graph, built from the low error rate short reads, to resolve repeats and loops/bulges within the graph. Therefore we would expect the resulting graph to have a similar error rate to an assembly generated from Illumina short reads alone. Illumina short reads are known to have a very low error rate and have become the tool of choice for calling high confidence SNPs in a number of recent resequencing studies (including Croucher 2011, Croucher 2015, Aanensen 2016, Holden 2013).
However, the question of assembly errors relative to the 'true' sequence is an important one and we are unaware of any direct comparisons of a 'read-threaded' genome to identify the potential error rate. In order to address this we took a mapping based approach using the low-error rate short reads independently. The short reads were mapped against USA300_FPR3757 and stringent parameters were used to identify only high confidence SNPs. These mapping based SNPs were compared to the list of SNPs generated by comparing the hybrid assembly to USA300_FPR757. Excluding SNPs found in regions exclusive to USA300 or repeat regions (which are notoriously problematic for mapping based approaches) 108/114 (92.1%) comparable SNPs were supported by this analysis. On visual investigation of the BAM file 6/9 of the unsupported SNPS were identified as being well supported variants with 100% read support that had been filtered based on significant strand bias caused by lower than average coverage (having only one supporting read on the reverse strand at 8x coverage). The remaining 3 SNPs (2.6%) were unsupported by mapping and may represent assembly error. Furthermore mapping of the reads to the genome allowed us to identify the number of reads supporting the edge of a large genomic structural variant. All structural variants were highly supported by 8-10x coverage of long reads and >25x coverage of short reads. The exception to this was one edge of the translocation event which only had moderate coverage with long reads (3-4x) but high coverage with short reads. In addition to this short reads were mapped to MHO_001. No indels were called using either samtools/GATK or pindel. 5 SNPs were called, 3 of which represented the SNPs unsupported by mapping to USA300 above. The remaining 2 were present in repeat regions.
We have added a spreadsheet of the comparison of SNPs and structural variant analysis as Supplementary Table 1. We have added the following text to the manuscript:

Materials and Method - SNPs were called between the chromosome and reference genome using MAUVE [14]☐. SNPs were further confirmed by mapping short reads independently to USA300_FPR3757 and calling variants. Mapping was performed using BWA, reads at indel sites were realigned using the GATK toolbox and SNPs were called using samtools [12,15]☐. The variant call file (VCF) was filtered for variants supported by a minimum read depth of 4 (minimum 2 per strand), >30 map quality, >50 average base quality, no significant strand bias and >75% of reads supporting the variant. Indels were additionally confirmed using pindel [16]☐. The VCF file was filtered to remove regions unique to MHO_001 or USA300_FPR3757. Repeat regions of >50bp, which are notoriously problematic for short read mapping, were identified using nucmer and removed from the comparison [17]☐ [Supplementary Table 1].

Results and Discussion - The chromosome showed minor differences to the USA300 reference genome USA300_FPR3757 including 155 SNP differences and the loss and gain of mobile genetic elements (Figure 2). In order to provide an independent confirmation of the 155 SNP differences identified by MAUVE between aligned regions of MHO_001 and USA300_FPR3757 the short reads were mapped to USA300_FPR3757 and variants were called using strict parameters. Of the 155 MAUVE SNPs 41 (26.5%) were present in repeat regions and excluded from the comparison. Of the remaining 114 SNPs, 111 (97.36%) were supported by short read mapping to USA300_FPR3757. The remaining 3 SNPs (2.6%) were unsupported. No indels were identified by short read mapping to MHO_001 by either GATK/samtools or pindel. In summary, of the 114

SNPs identified by MAUVE that could be robustly investigated by short read mapping 111 (97.4 %) were confirmed using low error rate short reads. Furthermore, the long and short read coverage support at the edge of each of the large structural variants in MHO_001 was 8-10x for nanopore reads, with the exception of the 3' edge of the transposed 13,356 bp insertion sequence (IS) which had a read coverage of 3x, compared to the genomic average of 6.8x coverage. The edge of each structural variant was supported by >25 short reads.

2) The authors declare that S. aureus MHO_001 was isolated from a case of asymptomatic nasal carriage (lines 53-54), however, during my review I was unable to find but no ethics any ethical nor methodological information regarding the collection of this sample and associated consent of patient, if necessary. Please add such information to the main text of the document.

This line was added in text “S. aureus strain MHO_001 was recovered in 2015 from asymptomatic nasal carriage via a standard nasal swab of a healthy individual with informed consent. ”

Minor points:

1) Along description of the MinION sequencing library the authors never declared the pore chemistry used for sequencing (lines 72-77). Please detail the R7.3 or R9 flow cells used to obtain nanopore data.

We used a Flow Cell Mark I R7.3. The text now reads “[samples were] loaded onto a MinION(TM) Flow Cell Mark I R7.3”.

2) In similar manner, please detail MinKNOW and Metrichor versions used to operate the MinION device and perform basecalling, respectively.

The text now reads “[samples were] loaded onto a MinION(TM) Flow Cell Mark I R7.3 on a MinION(TM) Mark I controlled by MinKNOW version 0.50.2.15 software (Oxford Nanopore, UK). Base calling was performed using Metrichor ONT Sequencing Workflow Software v1.19.0 with the Basecall_Barcoding workflow (Oxford Nanopore, UK)”.

3) In lines 81 and 89, accession numbers for read data must be presented primarily as "study accession" (PRJ numbers) and, secondarily, "sample accession" can be specified if needed (ERP or ERS numbers).

Both lines were corrected to “study accession PRJEB14152”.

4) In lines 111-113, describe what type of data is presented within and outside of parenthesis.

In order to address this I have moved the description of this data to the Results and Discussion as it was more pertinent for this section than the Materials and Method. The text now reads “There was a discrepancy observed between the coverage of short and long reads of plasmidic and chromosomal contigs (Figure 2, top and middle panels). The average chromosomal coverage was 49.6x (7.0 SD) with short read data and 6.8x (2.6 SD) with nanopore reads. The average short read coverage of plasmids A and B was 78.35 (8.9 SD) and 7302.04 (85.4 SD) respectively. This represents an coverage increase of 1.5- and 150-fold relative to the chromosome. The opposite trend was observed with long reads; plasmids A and B had and average coverage of 4.05 (2.0 SD) and 2.9 (1.7 SD) respectively, which represents a 40% and 60% decrease in coverage relative of the chromosome. In addition to this the smaller of the two plasmids was only intermittently covered by nanopore reads”.

5) Change the expression "close genome" to "complete genome" all across the text.

Modified throughout the manuscript.

6) Split the Figure 2 into panels A, B, C, D, etc, and make description in the legend to better understand the plots presented. Define information shown in all axes.

Figure 2 has been split into panels A, B and C and the figure legend has been revised for clarity. The legend now reads:

Figure 2. Alignment of MHO_001 chromosome (A), plasmid A (B) and plasmid B (C) to the USA300_FPR3757 genome and reference plasmids alongside long and short read coverage. The bottom panels show alignments between MHO_001 and the reference sequences. Contiguous sequences are shown by connecting red lines and inversions are depicted in blue. Coding sequences (CDS) are annotated as blue rectangles with the exception of ribosomal RNA operons which are represented by red rectangles. Those above the line represent open reading frames on the forward strand and those under the line on the reverse strand. Notable mobile genetic elements or genomic features are annotated. A scale bar in basepairs (bp) is present underneath each sequence. The middle panels represent per base read coverage of short reads across the MHO_001 genome. The data was binned every 1000 bp. The y-axis, representing per bin read coverage, has been constrained to 200, 350 and 8000 reads per bin for the MHO_001 chromosome, plasmid A and plasmid B respectively. The top panel represents the per base read coverage of nanopore long reads across the MHO_001 genome. The data was binned every 1000 bp. The y-axis, representing per bin read coverage, has been constrained to 20 reads per bin for each contig.

7) Unless massive sequencing technologies deliver error-free data and the assembly algorithms evolve towards complete accuracy it cannot be possible to tell "...identification of correct assembly..." (lines 163-165). I suggest to modify this statement to "...large assemblies with less uncertainties..." that results more adequate to the current development state of sequencing technologies.

The text now reads “However, the clear benefit of hybrid sequencing is that it allows for the generation of larger assemblies with less uncertainties than by using a single sequencing technology preferentially over another”.

8) In line 121, change MINion to "MinION". Additionally, MinION is a trade mark of the Oxford Nanopore Technologies (ONT), therefore, it must be cited always across the document as MinION(TM).

Modified throughout the manuscript.
