# Supplementary material for: The use of Oxford Nanopore native barcoding for complete genome assembly
Source: Gigascience. 2017 Feb 24;6(3):1–6. doi: 10.1093/gigascience/gix001 (PMC5467021; doi:10.1093/gigascience/gix001)
Supplement: Response_to_reviewer_comments_Revision_1.pdf [file gix001_response_to_reviewer_comments_revision_1.pdf]

Response to reviewers

We would like to again thank the reviewers for taking time to provide detailed and insightful responses to the manuscript. We are confident that we have addressed the only substantial concern, raised by Reviewer 2, by confirming the structural variants in MHO_001 by PCR and Sanger sequencing. Minor additional modifications have been made to the manuscript to address the other suggestions made. We hope that you share our view that the additional work included as supplementary material, provides convincing support for the complete genome sequence presented in the manuscript, and has thus improved the manuscript.

We have attached a copy of this response with figures embedded for clarity as Response_to_Reviewers.pdf in the Supplementary Material.

Revisions

Reviewer #1: The authors present an improved manuscript and all my major compulsory revisions were taken into account.

None.

Reviewer #2: Once I've read the reviewed version of the manuscript entitled, I have observed that authors have incorporated new information making the work more detailed. Although the authors argued properly the presence of SNPs by the high coverage accumulated and particularly supporting such genetic variants, I'm still requesting the confirmation of larger structural features found in the MHO_001 strain by gold standard methods. Particularly, the lack of of the SAPI5 island (line 183) and the expansion of the tRNA cluster (lines 192-196) would be feasible to demonstrate by simple PCR reactions on the original genomic DNA obtained from MHO_001 targeting these regions themselves as well as the contiguous sequences.

When draft genomes are published they normally appear compiled in dozens of scaffolds and contigs assembled from second generation sequencing technologies, and not matter how they are arranged because the assembly is not finished, therefore expected big changes in their definitive and complete fashion. Notwithstanding, the authors proposed the publication of a complete genome by hybrid approaches including those producing large and low-quality DNA reads, and they reported a detailed list of new features present in their isolate MHO_001 that could be clinically of relevance. Therefore, gold standard approaches such as PCR and/or Sanger sequencing must be employed to confirm the results stated here with the aim to give a valid starting point for future studies, in case the author want to set this workflow as a routine for the general audience. Notice that the draft and even complete genomes publicly available in most common database are highly susceptible to contain a large proportion of misassemblies (Muggli et al., Bioinformatics 2015;31:i80-8), consequently, if the author want to literally "demonstrate that MinION(TM) reads, multiplexed using native barcoding, can be used in combination with short-read data, to fully complete a bacterial genome", they have to demonstrate by conventional methods that their assembly is right and the structural variations found are real and not product of DNA reads and assembly artifacts.

We thank the referee for this useful suggestion and agree that this extra analysis is necessary if the workflow is to be widely adopted as hoped. In order to address this valid concern we investigated the expansion of the tRNA cluster and the lack of SAPI5 using PCR and Sanger sequencing, as suggested (Supplementary Analysis). MHO_001 and USA300 strain JE2 were used to compare presence/absence of SAPI5 and an expanded tRNA cluster. JE2 is a USA300 clone strain with 11 SNP differences when mapped to USA300_FPR3757 [1]. In order to identify the expansion of the tRNA island, primers were designed flanking this region. In order to test for the presence of SAPI5 four primers were designed; two primers flanking SaPI5 and two internal to SaPI5 (Supplementary Analysis Figure 1). By using the primers in three pairs, designated A (Primers 1+2), B (Primers 1,4) and C (Primers 3+4), we were able to determine if the island was present or absent. Pairs A and C amplified regions flanking SaPI5, starting from the flanking regions and extending towards the centre of SAPI5, which will only amplify if SAPI5 is present in that region of the

genome. Primer Pair B combined the primers of pairs A and C that were external to SAPI5 (Primers 1+4), and amplified between these flanking regions. Due to the size of SAPI5, amplification could only occur if the island was not present (Supplementary Analysis Figure 1).
The PCR amplicons were sequenced by Sanger sequencing (Eurofins). The PCR sequencing analysis confirmed that SAPI5 was not present in MH0_001 but present in JE2. Additionally, the tRNA island was found to be expanded in both MH0_001 and JE2 (Supplementary Analysis Figure 2). This confirmed that the assembly of MH0_001 as described in the main manuscript was correct. This also indicated that either the tRNA island was truncated in the strain representing the reference genome or the reference sequence is in error. In addition to this a very faint band was observed for primer pair B in the JE2 strain, perhaps indicating that SAPI5 is intermittently lost at low levels during culture or non-specific priming. However, we stress that these uncertainties only apply to the reference strain we used, which is a slight variant of the reference strain from which the reference genome sequence used in the paper was derived. These extra experiments are entirely consistent with out assembly of the the query MH0_001 strain. In conclusion, confirmation of two structural variants indicated that the complete genome sequences of MHO_001 was correct. Moreover, it is possible that our procedure provided a more accurate representation of genome structure of USA300 than the USA300_FPR3757 reference genome, although as we do not have the exact isolate from which this genome was generated it is not possible to confirm this.

Minor revisions

1) The authors should rename the "2D fail reads" because the audience could confound these with the 1D reads (which are 2D failed reads).

We agree with the reviewer that we should strive for maximum clarity within the manuscript. However, the “2D pass read” and “2D fail read” is consistent with the terminology adopted by both the manufacturer and by Karlsson et al. and Ip et al. [2,3] although Oikonomopoulos et al. referred to read group as 'high' and 'low' quality [4]☐. We were concerned that if we were to coin a new term for 2D fail reads then it maybe more confusing to the readership. Although we have maintained this terminology, we have clarified the description of the reads used and the terminology in the revised manuscript:
The paragraph “MinION(TM) 2D pass reads were demultiplexed by the Metrichor work flow. 2D reads that failed the sample QC were demultiplexed using an in-house script (FilterBarcodes.pl).” was changed to read “MinION(TM) 2D reads were filtered into pass and fail reads by the Metrichore basecaller, hereafter these two categories of reads will be referred to as “2D pass” and “2D fail” reads, following the terminology adopted by the manufacturer and used in Karlsson et al. and Ip et al. [2,3]. These are equivalent to the “high quality” and “low quality” read groups from Oikonomopoulos et al. [4]☐. MinION(TM) 1D reads were not used for this analysis. The 2D fail reads, those which did not pass the basecaller quality threshold, were demultiplexed using an in-house script (FilterBarcodes.pl).”

2) In the line 102, the authors cite that Illumina reads were trimmed to a minimum quality score of Q15. Is this right? The threshold for selection high quality Illumina reads normally is Q20 that means a per-base error probability of 1/100 (1% of miscalling probability). Lower scores in DNA reads can induce sequence errors or artificial SNPs.

We agree that Q20 is more prudent when assembling samples with good coverage. However, stricter quality thresholds reduce the number of paired end reads for assembly and we must necessarily weigh the trade-off between quantity and quality. Additionally, SPAdes also performs k-mer frequency based error correction using BayesHammer by default, which we did not disable. Therefore, it is likely that the hard threshold of Q15 which we used for initial trimming was not representative of the per-base error rate of the reads used for assembly, which would be lower after error correction.
We reassembled the genome using reads filtered to a quality score of 20 in order to assess whether read quality or coverage had a measurable impact on the assembly. This led to a reduction in the number of short paired reads passed to SPAdes from 439,480 to 393,649 (9%). The resulting assembly recreated the previously described four contigs. The two contigs corresponding to the plasmid were 100% identical. The chromosomal contig contained a 37 nucleotide differences (using MAUVE) to the Q15 contigs, of which 28

were in repeat regions which we have previously highlighted as being difficult to assemble, such as the spacer regions in ribosomal RNA operons. Of the remaining differences, 6 are were tightly clustered and the remaining 3 were dispersed throughout the genome.
We compared the Q15 and Q20 assemblies to USA300_FPR3757 (excluding SAPI5 and the novel bacteriophage in MHO_001). Of the 9 base differences between the Q15 and Q20 assemblies 7 of them were unique to the Q20 assembly and likely indicate assembly error in the Q20 assembly, one was unique to the Q20 assembly and one was a difference between the Q20 and Q30 assemblies in a region that was not present in USA300_FPR757.
In conclusion, it is often prudent to use higher quality thresholds when trimming reads for assembly. In this instance the more conservative threshold introduced a number of SNPs that were not present in either the original assembly or the reference genome. It is likely that this is due to the reduced number of reads passed to the assembler which had a negative impact on error correction and assembly. The assembly presented in the original manuscript has fewer SNPs relative to the to the reference genome and likely represents a more accurate representation of the genome of MHO_001.
The following line was added to the materials and method - “The absence of SAPI5 in MHO_001 and expansion of the tRNA island at 554,826 were confirmed using PCR and Sanger sequencing (Supplementary Analysis).”.

3) The authors must change "Oxford Nanopore, UK" by "Oxford Nanopore Technologies, UK" along the manuscript, and defined properly the "ONT" acronym in line 61 by stating this immediately after its meaning.

"Oxford Nanopore, UK" was changed to "Oxford Nanopore Technologies, UK" throughout the document and the abbreviation was added after the first usage in the main text.

4) The MinION device used is distributed and known as the MinION(TM) MkI and not "MarkI".

Mark 1 has been changed to Mk1 throughout the manuscript.

5) The "hit length" concept should be change to the "alignment length" (in terms of the proportion of DNA read aligned) a more adequate and common term used in this type of analysis. Please, do this in the main text and tables.

The term “hit length” has been changed to “alignment length” throughout the manuscript. Additional small changes have been made to the text to improve clarity.

References

1. Kennedy AD, Otto M, Braughton KR, Whitney AR, Chen L, Mathema B, et al. Epidemic community-associated methicillin-resistant Staphylococcus aureus: recent clonal expansion and diversification. Proc. Natl. Acad. Sci. National Acad Sciences; 2008;105:1327–32.
2. Karlsson E, Lärkeryd A, Sjödin A, Forsman M, Stenberg P. Scaffolding of a bacterial genome using MinION nanopore sequencing. Sci. Rep. Nature Publishing Group; 2015;5:11996.
3. Ip CLC, Loose M, Tyson JR, de Cesare M, Brown BL, Jain M, et al. MinION Analysis and Reference Consortium: Phase 1 data release and analysis. F1000Research. 2015;4:1–35.
4. Oikonomopoulos S, Wang YC, Djambazian H, Badescu D, Ragoussis J. Benchmarking of the Oxford Nanopore MinION sequencing for quantitative and qualitative assessment of cDNA populations. Sci. Rep. Nature Publishing Group; 2016;6:31602.
