# Supplementary material for: The use of Oxford Nanopore native barcoding for complete genome assembly
Source: Gigascience. 2017 Feb 24;6(3):1–6. doi: 10.1093/gigascience/gix001 (PMC5467021; doi:10.1093/gigascience/gix001)
Supplement: Response_to_reviewer_comments_Revision_2.pdf [file gix001_response_to_reviewer_comments_revision_2.pdf]

Dear Editors,

The requested information has been added to the end of the manuscript and to the GitHub repository. The information added has been summarised below.

Data Availability
The dataset supporting the conclusions of this article is available in the European Nucleotide Archive repository under project number PRJEB14152. Further supporting data is also available from the GigaScience GigaDB repository [22].

Availability and requirements
Project name: MHO_001 hybrid read assembly and analysis
Project home page: https://github.com/SionBayliss/MHO_analysis
Operating system: Unix
Programming language: R, perl
Other requirements: Dependencies include Samtools (>=1.18), Trimmomatic, SPAdes v3.6.1, BWA (0.7.5a-r405), BioPerl, MAUVE, BLASR, prokka, Tablet/Artemis
License: GNU GPL v3

Additional reference added:

22. Bayliss SC, Hunt VL, Yokoyama M, Thorpe HA, Feil EJ. Supporting data for "The use of Oxford Nanopore native barcoding for complete genome assembly". GigaScience Database. 2016. http://dx.doi.org/10.5524/100269.
