# Supplementary material for: The use of Oxford Nanopore native barcoding for complete genome assembly
Source: Gigascience. 2017 Feb 24;6(3):1–6. doi: 10.1093/gigascience/gix001 (PMC5467021; doi:10.1093/gigascience/gix001)
Supplement: Reviewer_1_Report_Original_Submission.pdf [file gix001_reviewer_1_report_original_submission.pdf]

Reviewer Report

Title: "The use of Oxford Nanopore native barcoding for complete genome assembly"

Version: Original Submission **Date:** 6/16/2016

Reviewer name: Jean-Marc Aury

Reviewer Comments to Author:

The manuscript "The use of Oxford Nanopore native barcoding for complete genome assembly" by Bayliss et al. describes a methodology dedicated to genome assembly of small genomes by combining the MinION device with the Illumina sequencing technology. Several studies have already reported the use of the Oxford Nanopore technology for genome assembly, as mentioned by the authors. Here, authors take advantage of the possibility of multiplexing libraries on a single sequencing run. The manuscript describes the sequencing data and the minor differences between the USA300 reference genome and the MHO_001 strain. The assembly method is classical and has already been used in Risse et al. (GigaScience 2015) to assemble the Bacteroides fragilis genome. My main concern about the manuscript is related to the weak description and analysis of the multiplexed data. In addition, the impact is limited because the method used and the results obtained improve slightly the state of the art for nanopore-based assembly.

Major Compulsory Revisions

1) I recommend the authors to give a better insight of the usage of barcodes. Several questions are emerging, like for example the accuracy of the demultiplexing process (given the high error rate of nanopore reads), the fraction of cross-contamination (reads that are assigned to a wrong sample). These two informations could affect the required sequencing coverage.

2) The samples used for the pooled library are not defined. Authors should add a full description of the dataset. I couldn't find it at the given URL: http://porecamp.github.io

3) Please describe in more details the illumina data. Authors argue that the sequencing was performed on MiSeq and HiSeq platforms, which dataset was used to perform the genome assembly with Spades? What is the read length?

4) Please describe in more details how splitbarcodes.py performs the demultiplexing step.

Minor Essential Revisions

1) Line 96: Metrichore instead of Metrichor

2) Line 104: "…BLAST similarity with with previously…"

3) Line 165: "…lost using one method sequencing method preferentially…"

4) Line 100: Is Spades output a single contig with the same circularization point as the reference genome? Was this region correctly assembled?

Jean-Marc Aury

Level of Interest

Please indicate how interesting you found the manuscript: An article of limited interest

Quality of Written English

Please indicate the quality of language in the manuscript: Acceptable

Declaration of Competing Interests

Please complete a declaration of competing interests, considering the following questions:

- Have you in the past five years received reimbursements, fees, funding, or salary from an organisation that may in any way gain or lose financially from the publication of this manuscript, either now or in the future?
- Do you hold any stocks or shares in an organisation that may in any way gain or lose financially from the publication of this manuscript, either now or in the future?
- Do you hold or are you currently applying for any patents relating to the content of the manuscript?
- Have you received reimbursements, fees, funding, or salary from an organization that holds or has applied for patents relating to the content of the manuscript?
- Do you have any other financial competing interests?
- Do you have any non-financial competing interests in relation to this paper?

If you can answer no to all of the above, write 'I declare that I have no competing interests' below. If your reply is yes to any, please give details below.

I declare that I have no competing interests; however I should mention that I am part of the MinION® Access Programme (MAP).

I agree to the open peer review policy of the journal. I understand that my name will be included on my report to the authors and, if the manuscript is accepted for publication, my named report including any attachments I upload will be posted on the website along with the authors' responses. I agree for my report to be made available under an Open Access Creative Commons CC-BY license

(http://creativecommons.org/licenses/by/4.0/). I understand that any comments which I do not wish to be included in my named report can be included as confidential comments to the editors, which will not be published.

I agree to the open peer review policy of the journal
