# Supplementary material for: The use of Oxford Nanopore native barcoding for complete genome assembly
Source: Gigascience. 2017 Feb 24;6(3):1–6. doi: 10.1093/gigascience/gix001 (PMC5467021; doi:10.1093/gigascience/gix001)
Supplement: Reviewer_2_Report_Original_Submission.pdf [file gix001_reviewer_2_report_original_submission.pdf]

Reviewer Report

Title: "The use of Oxford Nanopore native barcoding for complete genome assembly"

Version: Original Submission **Date:** 6/16/2016

Reviewer name: Alfonso Benitez-Paez, Ph.D.

Reviewer Comments to Author:

The authors report the complete genome assembly of the Staphylococcus aureus MHO_001 strain using hybrid approaches based on analysis of Illumina and MinION data. Although such type of analysis can be found regularly in the scientific literature, the authors put special attention to the usage of a very small subset of MinION reads to get a complete genome assembly of their pathogenic isolate. MinION data was combined with a the large amount of Illumina reads, obtained by massive and parallel sequencing in MiSeq and HiSeq platforms, to produce three major scaffolds representing the bacterial chromosome of S. aureus MHO_001 and two mobile elements consisting of two plasmids SAP046A.

In my opinion, this work represent an interesting workflow to help in the achievement of valuable information regarding the complete sequencing of bacterial genomes, where the genome structure complexity and the limitations in terms of read length of the second generation of sequencing technologies constitute the major issues. As a consequence, I recommend this manuscript for publication in GigaScience journal once the following minor and major changes have been incorporated to the main text and supporting material:

Major points:

1) MinION reads are characterized by their high rate of errors that can reach up to 40% in 1D reads. The authors declare usage of only 2D reads for the hybrid genome assembly, however, even in this type of reads the per-base accuracy reaches 85% as the most (for R7.3 chemistry). With the above information and the low number of MinION reads used for the manuscript general aim, I would suggest to perform some additional analysis in order to validate the strategy presented. This will imply to corroborate by Sanger sequencing, when technically possible, the genetic variants and chromosome rearrangements found in the MHO_001 strain in comparison with the USA300.

2) The authors declare that S. aureus MHO_001 was isolated from a case of asymptomatic nasal carriage (lines 53-54), however, during my review I was unable to find but no ethics any ethical nor methodological information regarding the collection of this sample and associated consent of patient, if necessary. Please add such information to the main text of the document.

Minor points:

1) Along description of the MinION sequencing library the authors never declared the pore chemistry used for sequencing (lines 72-77). Please detail the R7.3 or R9 flow cells used to obtain nanopore data.

2) In similar manner, please detail MinKNOW and Metrichor versions used to operate the MinION device and perform basecalling, respectively.

3) In lines 81 and 89, accession numbers for read data must be presented primarily as "study accession" (PRJ numbers) and, secondarily, "sample accession" can be specified if needed (ERP or ERS numbers).

4) In lines 111-113, describe what type of data is presented within and outside of parenthesis.

5) Change the expression "close genome" to "complete genome" all across the text.

6) Split the Figure 2 into panels A, B, C, D, etc, and make description in the legend to better understand the plots presented. Define information shown in all axes.

7) Unless massive sequencing technologies deliver error-free data and the assembly algorithms evolve towards complete accuracy it cannot be possible to tell "...identification of correct assembly..." (lines 163-165). I suggest to modify this statement to "...large assemblies with less uncertainties..." that results more adequate to the current development state of sequencing technologies.

8) In line 121, change MINion to "MinION". Additionally, MinION is a trade mark of the Oxford Nanopore Technologies (ONT), therefore, it must be cited always across the document as MinION(TM).

Level of Interest

Please indicate how interesting you found the manuscript: An article whose findings are important to those with closely related research interests

Quality of Written English

Please indicate the quality of language in the manuscript: Acceptable

Declaration of Competing Interests

Please complete a declaration of competing interests, considering the following questions:

- Have you in the past five years received reimbursements, fees, funding, or salary from an organisation that may in any way gain or lose financially from the publication of this manuscript, either now or in the future?

- Do you hold any stocks or shares in an organisation that may in any way gain or lose financially from the publication of this manuscript, either now or in the future?
- Do you hold or are you currently applying for any patents relating to the content of the manuscript?
- Have you received reimbursements, fees, funding, or salary from an organization that holds or has applied for patents relating to the content of the manuscript?
- Do you have any other financial competing interests?
- Do you have any non-financial competing interests in relation to this paper?

If you can answer no to all of the above, write 'I declare that I have no competing interests' below. If your reply is yes to any, please give details below.

I have received funding in the past in term of reagents and consumables from Oxford Nanopore Technologies, who has the ownership of MinION sequencing platform.

I agree to the open peer review policy of the journal. I understand that my name will be included on my report to the authors and, if the manuscript is accepted for publication, my named report including any attachments I upload will be posted on the website along with the authors' responses. I agree for my report to be made available under an Open Access Creative Commons CC-BY license (http://creativecommons.org/licenses/by/4.0/). I understand that any comments which I do not wish to be included in my named report can be included as confidential comments to the editors, which will not be published.

I agree to the open peer review policy of the journal
