# Supplementary material for: The use of Oxford Nanopore native barcoding for complete genome assembly
Source: Gigascience. 2017 Feb 24;6(3):1–6. doi: 10.1093/gigascience/gix001 (PMC5467021; doi:10.1093/gigascience/gix001)
Supplement: Reviewer_2_Report_Revision_1.pdf [file gix001_reviewer_2_report_revision_1.pdf]

Reviewer Report

Title: "The use of Oxford Nanopore native barcoding for complete genome assembly"

Version: Revision 1 **Date:** 9/6/2016

Reviewer name: Alfonso Benitez-Paez, Ph.D.

Reviewer Comments to Author:

Once I've read the reviewed version of the manuscript entitled, I have observed that authors have incorporated new information making the work more detailed. Although the authors argued properly the presence of SNPs by the high coverage accumulated and particularly supporting such genetic variants, I'm still requesting the confirmation of larger structural features found in the MHO_001 strain by gold standard methods. Particularly, the lack of of the SAPI5 island (line 183) and the expansion of the tRNA cluster (lines 192-196) would be feasible to demonstrate by simple PCR reactions on the original genomic DNA obtained from MHO_001 targeting these regions themselves as well as the contiguous sequences.

When draft genomes are published they normally appear compiled in dozens of scaffolds and contigs assembled from second generation sequencing technologies, and not matter how they are arranged because the assembly is not finished, therefore expected big changes in their definitive and complete fashion. Notwithstanding, the authors proposed the publication of a complete genome by hybrid approaches including those producing large and low-quality DNA reads, and they reported a detailed list of new features present in their isolate MHO_001 that could be clinically of relevance. Therefore, gold standard approaches such as PCR and/or Sanger sequencing must be employed to confirm the results stated here with the aim to give a valid starting point for future studies, in case the author want to set this workflow as a routine for the general audience. Notice that the draft and even complete genomes publicly available in most common database are highly susceptible to contain a large proportion of misassemblies (Muggli et al., Bioinformatics 2015;31:i80-8), consequently, if the author want to literally "demonstrate that MinION(TM) reads, multiplexed using native barcoding, can be used in combination with short-read data, to fully complete a bacterial genome", they have to demonstrate by conventional methods that their assembly is right and the structural variations found are real and not product of DNA reads and assembly artifacts.

Minor points:

1) The authors should rename the "2D fail reads" because the audience could confound these with the 1D reads (which are 2D failed reads).

2) In the line 102, the authors cite that Illumina reads were trimmed to a minimum quality score of Q15. Is this right? The threshold for selection high quality Illumina reads normally is Q20 that means a per-base error probability of 1/100 (1% of miscalling probability). Lower scores in DNA reads can induce sequence errors or artificial SNPs.

3) The authors must change "Oxford Nanopore, UK" by "Oxford Nanopore Technologies, UK" along the manuscript, and defined properly the "ONT" acronym in line 61 by stating this immediately after its meaning.

4) The MinION device used is distributed and known as the MinION(TM) MkI and not "MarkI".

5) The "hit length" concept should be change to the "alignment length" (in terms of the proportion of DNA read aligned) a more adequate and common term used in this type of analysis. Please, do this in the main text and tables.

Level of Interest

Please indicate how interesting you found the manuscript: An article whose findings are important to those with closely related research interests

Quality of Written English

Please indicate the quality of language in the manuscript: Acceptable

Declaration of Competing Interests

Please complete a declaration of competing interests, considering the following questions:

- Have you in the past five years received reimbursements, fees, funding, or salary from an organisation that may in any way gain or lose financially from the publication of this manuscript, either now or in the future?
- Do you hold any stocks or shares in an organisation that may in any way gain or lose financially from the publication of this manuscript, either now or in the future?
- Do you hold or are you currently applying for any patents relating to the content of the manuscript?
- Have you received reimbursements, fees, funding, or salary from an organization that holds or has applied for patents relating to the content of the manuscript?
- Do you have any other financial competing interests?
- Do you have any non-financial competing interests in relation to this paper?

If you can answer no to all of the above, write 'I declare that I have no competing interests' below. If your reply is yes to any, please give details below.

I declare that I have no competing interests

I agree to the open peer review policy of the journal. I understand that my name will be included on my report to the authors and, if the manuscript is accepted for publication, my named report including any attachments I upload will be posted on the website along with the authors' responses. I agree for my report to be made available under an Open Access Creative Commons CC-BY license (http://creativecommons.org/licenses/by/4.0/). I understand that any comments which I do not wish to be included in my named report can be included as confidential comments to the editors, which will not be published.

I agree to the open peer review policy of the journal
