# Supplementary material for: The use of Oxford Nanopore native barcoding for complete genome assembly
Source: Gigascience. 2017 Feb 24;6(3):1–6. doi: 10.1093/gigascience/gix001 (PMC5467021; doi:10.1093/gigascience/gix001)
Supplement: Supplement Files [file gix001_supp.zip › DataDescription.docx]

Dear Editor,

Please find attached our manuscript entitled, “The use of Oxford Nanopore native barcoding for complete genome assembly” for consideration for publication in Gigascience. This paper represents, to the best of our knowledge, the first account of a bacterial genome being closed using the native barcoding protocol for Oxford Nanopore Technologies MinION DNA sequencer. The genome of Staphylococcus aureus strain MHO001 (USA300) was closed using a hybrid assembly of nanopore long reads with matching Illumina short reads. The finished genome showed a very low level of divergence to the nearest reference genome, excepting the loss or gain of mobile genetic elements, consistent with low error rates.

A key finding of the work was that considerably fewer long reads were necessary to produce a high quality closed genome than in previous publications, pointing to the ability to assemble multiplexed MinION sample as an extremely cost-effective solution. The methods described have direct applications for epidemiological and evolutionary studies, and are likely to be of great interest to the readership of Gigascience. The protocol used to assemble the genome has been described in detail and made available on GitHub, along with the dataset, for use as an example for hybrid assembly.

This manuscript describes original work and is not under consideration by any other journal. All authors approved the manuscript and this submission. Thank you for receiving our manuscript and considering it for review. We appreciate your time and look forward to your positive response.

Yours Faithfully,

Dr Sion Bayliss (on behalf of all co-authors)
